# Mechanism of Intramembrane Cleavage of Alcadeins by γ-Secretase

**DOI:** 10.1371/journal.pone.0062431

**Published:** 2013-04-26

**Authors:** Yi Piao, Ayano Kimura, Satomi Urano, Yuhki Saito, Hidenori Taru, Tohru Yamamoto, Saori Hata, Toshiharu Suzuki

**Affiliations:** 1 Laboratory of Neuroscience, Graduate School of Pharmaceutical Sciences, Hokkaido University, Sapporo, Japan; 2 Laboratory of Neural Cell Biology, Graduate School of Pharmaceutical Sciences, Hokkaido University, Sapporo, Japan; 3 Creative Research Institute, Hokkaido University, Sapporo, Japan; Nathan Kline Institute and New York University School of Medicine, United States of America

## Abstract

**Background:**

Alcadein proteins (Alcs; Alcα, Alcβand Alcγ) are predominantly expressed in neurons, as is Alzheimer's β-amyloid (Aβ) precursor protein (APP). Both Alcs and APP are cleaved by primary α- or β-secretase to generate membrane-associated C-terminal fragments (CTFs). Alc CTFs are further cleaved by γ-secretase to secrete p3-Alc peptide along with the release of intracellular domain fragment (Alc ICD) from the membrane. In the case of APP, APP CTFβ is initially cleaved at the ε-site to release the intracellular domain fragment (AICD) and consequently the γ-site is determined, by which Aβ generates. The initial ε-site is thought to define the final γ-site position, which determines whether Aβ40/43 or Aβ42 is generated. However, initial intracellular ε-cleavage sites of Alc CTF to generate Alc ICD and the molecular mechanism that final γ-site position is determined remains unclear in Alcs.

**Methodology:**

Using HEK293 cells expressing Alcs plus presenilin 1 (PS1, a catalytic unit of γ-secretase) and the membrane fractions of these cells, the generation of p3-Alc possessing C-terminal γ-cleavage site and Alc ICD possessing N-terminal ε-cleavage site were analysed with MALDI-TOF/MS. We determined the initial ε-site position of all Alcα, Alcβ and Alcγ, and analyzed the relationship between the initially determined ε-site position and the final γ-cleavage position.

**Conclusions:**

The initial ε-site position does not always determine the final γ-cleavage position in Alcs, which differed from APP. No additional γ-cleavage sites are generated from artificial/non-physiological positions of ε-cleavage for Alcs, while the artificial ε-cleavage positions can influence in selection of physiological γ-site positions. Because alteration of γ-secretase activity is thought to be a pathogenesis of sporadic Alzheimer's disease, Alcs are useful and sensitive substrate to detect the altered cleavage of substrates by γ-secretase, which may be induced by malfunction of γ-secretase itself or changes of membrane environment for enzymatic reaction.

## Introduction

The γ-secretase is comprised of four membrane proteins, presenilin 1 (PS1) or 2 (PS2), nicastrin (NCT), anterior pharynx defective 1 (APH-1), and presenilin enhancer 2 (PEN-2) [1]. PS functions as the catalytic unit of this aspartyl protease complex [2]. Prior to intramembrane cleavage of type I membrane proteins by γ-secretase, the substrate membrane proteins are subject to primary extracellular/intraluminal cleavage at the juxtamembrane region by a sheddase such as a disintegrin and metalloproteinase (ADAM) [3]. This primary cleavage is essential for the subsequent intramembrane γ-cleavage, although the exact regulation of intramembrane cleavage by γ-secretase remains unclear.

There are no distinct consensus amino acid sequences of γ-cleavage sites among over 60 different proteins reported as substrates of γ-secretase [1]. However, the molecular mechanisms of γ-cleavage of the Alzheimer's disease (AD)-related β-amyloid precursor protein (APP) and Notch have been well characterized [4], [5]. APP is cleaved by β-secretase (BACE) in addition to α-secretase (ADAM 10 and ADAM 17), and retains the C-terminal fragments, amyloidogenic CTFβ and amyloidolytic CTFα, in the membrane while secreting the large extracellular N-terminal fragments [3]. When CTFβ is further cleaved by γ-secretase, the AD-related amyloid β protein (Aβ) is generated, while metabolically labile p3 peptide is generated from CTFα by γ-cleavage. This intramembrane γ-cleavage of APP CTF occurs initially at ε-cleavage sites. ε-cleavage between Leu645 and Val646 of APP695 generates Aβ49, which is processed to generate Aβ46 and Aβ43, and γ-cleavage at a site between Val636 and Ile637 generates Aβ40, a major Aβ species. When alternative ε-cleavage occurs between Thr644 and Leu645, Aβ48, Aβ45, and Aβ42 are sequentially generated, and cleavage at Gly634 generates Aβ38. These Aβ peptides are generated by processing of every three to four amino acids from the initial ε-site by γ-secretase [6]–[10]. Therefore, based on these observations, the initial ε-cleavage site nearly defines the position of the final γ-cleavage site in APP.

If this procedure is basically common among type I membrane protein substrates, the first-determined ε-cleavage site is prerequisite to the generation of alternative products toward a specific γ-cleavage site. For example, the familial AD (FAD)-linked PS mutations are thought to alter the position of the first ε-site, which in turn contributes to changing the production ratio of γ-site cleaved products 11,12. In fact, FAD-linked mutations of PS1 increase the Aβ42/Aβ40 ratio in comparison to wild-type PS1 by increasing the alternative ε-site cleavage of APP CTFβ [13]. Alternatively, an alteration of γ-secretase function may not reach to major γ-site position and increase minor γ-cleavage, by which production of Aβ42 increases while Aβ38 generation decreases in APP [14], [15]. In this study, we explored whether the correlation between γ- and ε-cleavage sites is also common to the Alcadein family of proteins (Alcs): Alcα, Alcβ and Alcγ.

Alcs are encoded by independent genes and expressed largely in neurons [16]. Alcs are primarily cleaved by APP α-secretase to generate Alc CTFs, which are consequently cleaved by γ-secretase, like APP, to secrete a short peptide p3-Alc into cell media or cerebrospinal fluid (CSF), along with liberation of the intracellular cytoplasmic domain fragment Alc ICD [17]. Thus, the C-terminal amino acid residue of p3-Alc contains a γ-cleavage site of Alc, and the N-terminal amino acid residue of Alc ICD demonstrates an initial ε-cleavage site. We previously showed that cells expressing FAD-linked PS1 mutation demonstrated an altered ratio of γ-cleavage products, with the increase of minor γ-cleaved products (minor p3-Alc species) to major p3-Alc species in these cells reflected by the increase of Aβ42 (the minor species) to Aβ40 (the major species) [17]. Because the magnitude of alternative γ-cleavage of Alcs and APP to generate p3-Alcα, p3-Alcβ, p3-Alcγ, and Aβ was not equivalent in cells expressing FAD-linked PS1 mutants [17], we speculated that their mechanisms of intramembrane substrate cleavage by γ-secretase may differ, or their sensitivity to altered γ-secretase activity may differ. Furthermore, we reported the increase of a minor species, p3-Alcα38, in the CSF of sporadic AD (SAD) patients, suggesting that γ-secretase dysfunction occurs in some populations of SAD patients [18]. Thus, understanding the mechanism by which Alcs are cleaved by γ-secretase is important to gaining deeper insight into the pathogenesis of AD.

The γ-secretase was found to cleave Alcs (Alcα, Alcβ and Alcγ ) [19], and their γ-cleavage sites were determined as the C-termini of p3-Alcα, p3-Alcβ, and p3-Alcγ. These γ-cleavage sites were also demonstrated in human CSF [17], [18]. We previously reported that in human, the major p3-Alcα species is p3-Alcα35 with C-terminal cleavage at Thr851 (numbering for Alcα1 isoform), and the minor species is p3-Alcα38 with C-terminal cleavage at Ile854. The major p3-Alcβ species in human CSF is p3-Alcβ37 with C-terminal cleavage at Thr849, while the minor species is p3-Alcβ40 with C-terminal cleavage at Ile852. However, in cells expressing Alcβ, the major species is p3-Alcβ40, and the minor species is p3-Alcβ37. In both human CSF and cells expressing Alcγ, the major p3-Alcγ species is p3-Alcγ31 with C-terminal cleavage at Thr834, and the minor species is p3-Alcγ34 with C-terminal cleavage at Ile837 [17]. Determination of the Alcs γ-site was somewhat equivocal, because the major γ-site of p3-Alcβ differed between CSF and cultured cells [17]. Therefore, to explore the mechanism for intramembrane cleavage of Alcs in this study, we first determined the ε-cleavage sites of Alcs. We then analyzed the relationship between initial determination of ε-cleavage site and final γ-cleaved site.

## Results

### Determination of Intramembrane ε-cleaving Site of Alcadeins

**Figure 1:**
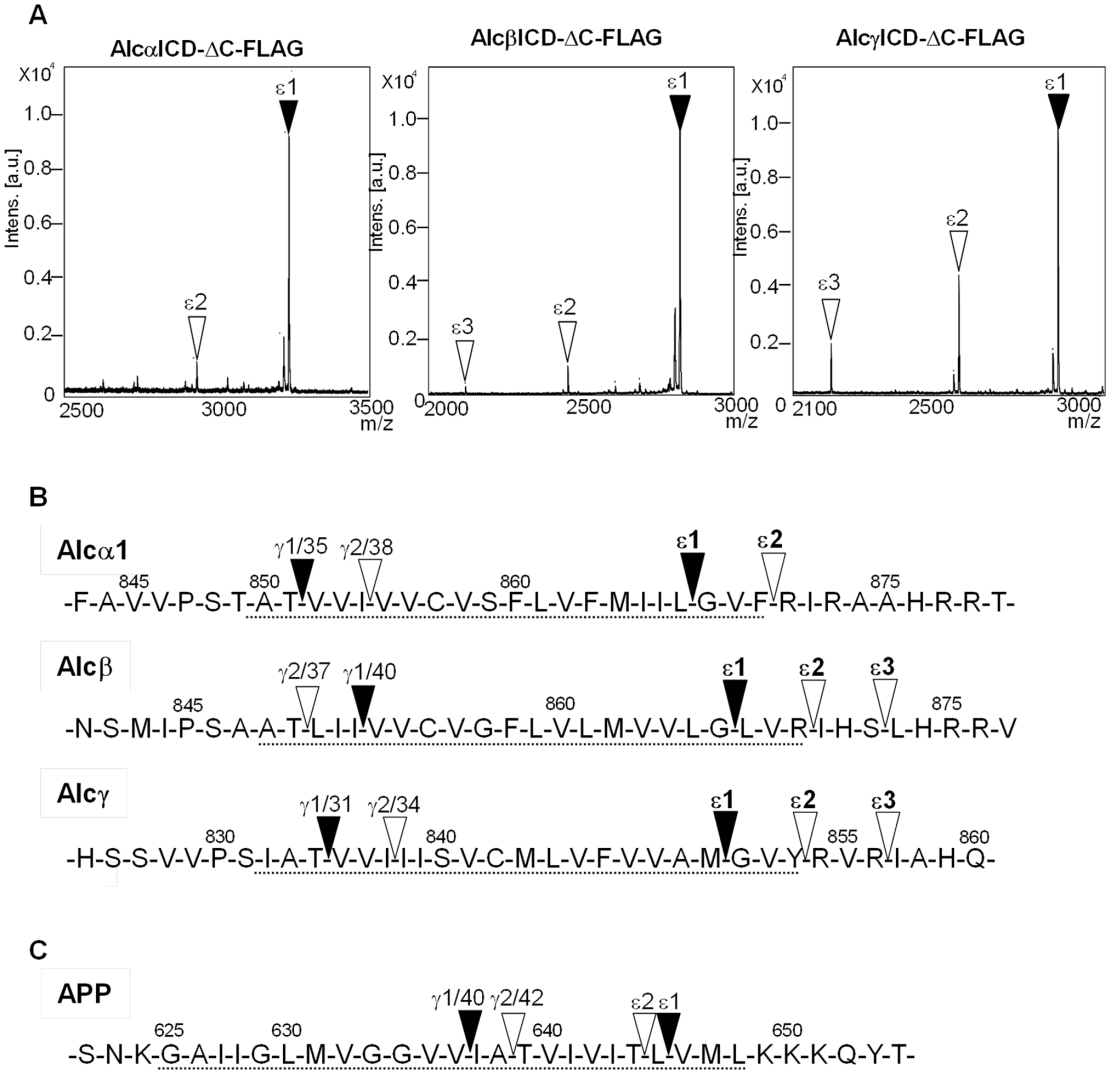
Determination of intramembrane ε-cleavage sites of Alcadeins. Representative mass spectra of Alc ICD-ΔC-FLAG generated by *in vitro* γ-secretase assay with membranes from HEK293 cells expressing Alc-ΔC-FLAG (**A**), and localization of ε-cleavage sites on the amino acid sequence (**B**), along with the comparison to γ- and ε-cleavage sites of APP (**C**). **A.** AlcαICD-ΔC-FLAG generated from Alcα-ΔC-FLAG (left), Alcβ ICD-ΔC-FLAG generated from Alcβ-ΔC-FLAG (middle), and Alcγ ICD-ΔC-FLAG generated from Alcγ-ΔC-FLAG (right). Closed arrowheads indicate the major product cleaved at the ε1 site, and open arrowheads indicate the minor products cleaved at the ε2 and ε3 sites. Amino acid sequence of Alc ICD-ΔC-FLAG was determined (**Fig. S3**). Other peaks, which are not indicated with arrowheads, are not products derived from Alc-ΔC-FLAG, because they are detectable in cells expressing an inactive/dominant-negative PS1 D385A mutant (**Fig. S2**). **B.** Amino acid sequence of human Alcα1, Alcβ and Alcγ (numbers indicate amino acid position, and the broken underline indicates putative transmembrane region). The major (ε1) and minor (ε2 and ε3) ε-cleavage sites are indicated, along with the previously identified major (γ1) and minor (γ2) γ-cleavage sites (13). In Alcα (upper), cleavage at γ1 generates p3-Alcα35, while cleavage at γ2 generates p3-Alcα38. Therefore, the γ1 cleavage site “γ1/35” and the γ2 cleavage site “γ2/38” are shown. Cultured cell lines generate p3-Alcα2N+35 and p3-Alcα2N+38, which possess two extra amino acids at the N terminal but have identical γ-cleavage sites to p3-Alcα35 and p3-Alcα38 in human CSF and are therefore considered the products cleaved at γ1 and γ2, respectively. In Alcβ (middle), cleavage at γ1 (γ1/40) generates p3-Alcβ40, while cleavage at γ2 (γ2/37) generates p3-Alcβ37. In cultured cell lines, “γ1/40” is the major γ-cleavage site, but “γ2/37” is the major site in human CSF. In Alcγ (lower), cleavage at the major cleavage siteγ1 (γ1/31) generates p3-Alcγ31, while cleavage at the minor site γ2 (γ2/34) generates p3-Alcγ34. Schematic pictures of protein constructs used in this study are shown in **Fig. S1.** **C.** Major and minor γ- and ε-cleavage sites of APP. The major γ1 (γ1/40) and minor γ2 (γ2/42) cleavage sites are shown. In APP, the major ε1 site largely defines the γ1 site to generate Aβ40, and the minor ε2 site promotes the γ2 site to generate Aβ42 or Aβ38.

To identify the ε-cleavage sites of Alcs, we determined the N-terminal amino acid of Alc ICD. HEK293 cells expressing AlcΔC-FLAG (**Fig. S1**) were subjected to treatment with the γ-secretase inhibitor DAPT (3,5-(Difuorophenyl)acetyl-L-alanyl-L-2-phenylglycine *t*-butyl ester) to accumulate membrane-associated Alc CTFΔC-FLAG, which is the C-terminal product of AlcΔC-FLAG cleaved by primary α-secretase. Membranes were prepared from the cells and then subjected to *in vitro* γ-secretase assay to facilitate the cleavage of Alc CTFΔC-FLAG. The generated intracellular Alc ICDΔC-FLAG was isolated by immunoprecipitation, and representative MS spectra are shown (**Fig. 1A**). In Fig. 1A, the signals indicated with arrowheads are γ-secretase-dependent products because cells expressing a dominant-negative PS1 mutant carrying D385A substitution did not generate these Alc ICD species (**Fig. S2**).

The molecular masses observed by TOF/MS analysis (**Fig. 1A**) were compared with expected values (**Table S1**), and the amino acid sequence was determined with matrix-assisted laser desorption ionization time-of-flight tandem mass spectrometry (MALDI-MS/MS) analysis (**Fig. S3**). The γ-cleavage site was also confirmed by MALDI-MS/MS analysis of p3-Alc secreted by cells expressing AlcΔC-FLAG as described [17]. The ε- and γ-cleavages observed upon *in vitro* membrane incubation were consistent with the cleavages observed in cells. The minor/major γ-cleavage ratio of p3-Alc generated by the *in vitro* γ-secretase assay with membrane coincided with the minor/major γ-cleavage ratio of p3-Alc secreted by cells, and the production levels of p3-Alc were very similar between the *in vitro* γ-secretase assay and cultured cells (**Fig. S4**).

In the *in vitro* γ-secretase assay with membrane prepared from cells expressing AlcαΔC-FLAG, Alcα truncated at Gly886 with C-terminal FLAG (**Fig. S1,** and amino acid sequence around the ε-sites shown in **Fig. 1B**) generated two Alcα ICD species, MW3238 (major ε1) and MW2934 (minor ε2), including the FLAG-tag. Therefore, the major Alcα ICD species possessed Gly868 and minor Alc ICD species possessed Arg871 at their N-termini (**Fig. 1A** **, left; Fig. S3A**). Cells expressing AlcαΔC-FLAG secreted major p3-Alcα2N+35 (γ1) and minor p3-Alcα2N+38 (γ2) species (**Fig. S4A**), indicating that the γ-cleavage of Alcα was not affected by truncation of the cytoplasmic region and addition of the FLAG tag (See **ref.** **13,** for “2N+”species that possess the same C-terminal γ-site to p3-Alcα35 (γ1) and p3-Alcα38 (γ2), respectively; however, “2N+” species were predominantly generated from cultured cells.).

Identical analyses were performed with AlcβΔC-FLAG truncated at Ile881 and AlcγΔC-FLAG truncated at Ile866 (**Fig. S1**). The major Alcβ ICD species demonstrated MW 2824 (ε1) with two minor species of MW 2455 (ε2) and MW 2118 (ε3) including the FLAG tag. Therefore, the major Alcβ ICD species possessed Leu867, and the minor Alcβ ICD species possessed Ile870 and Leu873, respectively, at their N-termini (**Fig. 1A** **, middle; Fig. S3B**). The major Alcγ ICD demonstrated MW 2946 (ε1) with two minor species of MW 2627 (ε2) and MW 2216 (ε3) including the FLAG tag. Therefore the major Alcγ ICD species has an N-terminal Gly851 residue, and the minor Alcγ ICD species possess Arg854 and Ile857, respectively, at their N termini (**Fig. 1A** **, right; Fig. S3C**). The γ-cleavage sites of AlcβΔC-FLAG and AlcγΔC-FLAG were confirmed by determination of the amino acid sequences of p3-Alcβ and p3-Alcγ (**Fig. S4B and C**).

These results show that γ-secretase first cleaves Alcα CTF between Leu867 and Gly868 (ε1) for a major product and between Phe870 and Arg871 (ε2) for a minor product. Alcβ CTF is first cleaved between Gly866 and Leu867 (ε1) for a major product and between Arg869 and 870Ile (ε2) or Ser872 and Leu873 (ε3) for minor products, and Alcγ CTF is first cleaved between Met850 and Gly851 (ε1) for a major product and between Tyr853 and Arg854 (ε2) or Arg856 and Ile857 (ε3) for minor products. Some minor ε-cleavage sites may be located outside of putative Alcs transmembrane domains. However, again, the ε-cleavages are due to γ-secretase because PS1 dominant negative mutant cannot cleave Alc ICD at these ε-sites (**Fig. S2**). The locations of Alcα, Alcβ, and Alcγ, γ- and ε-cleavage sites were then compared to those of APP (**Fig. 1B and C**).

### Relationship between Altered Cleavage at γ-sites and at ε-sites in Alcs and APP

**Figure 2:**
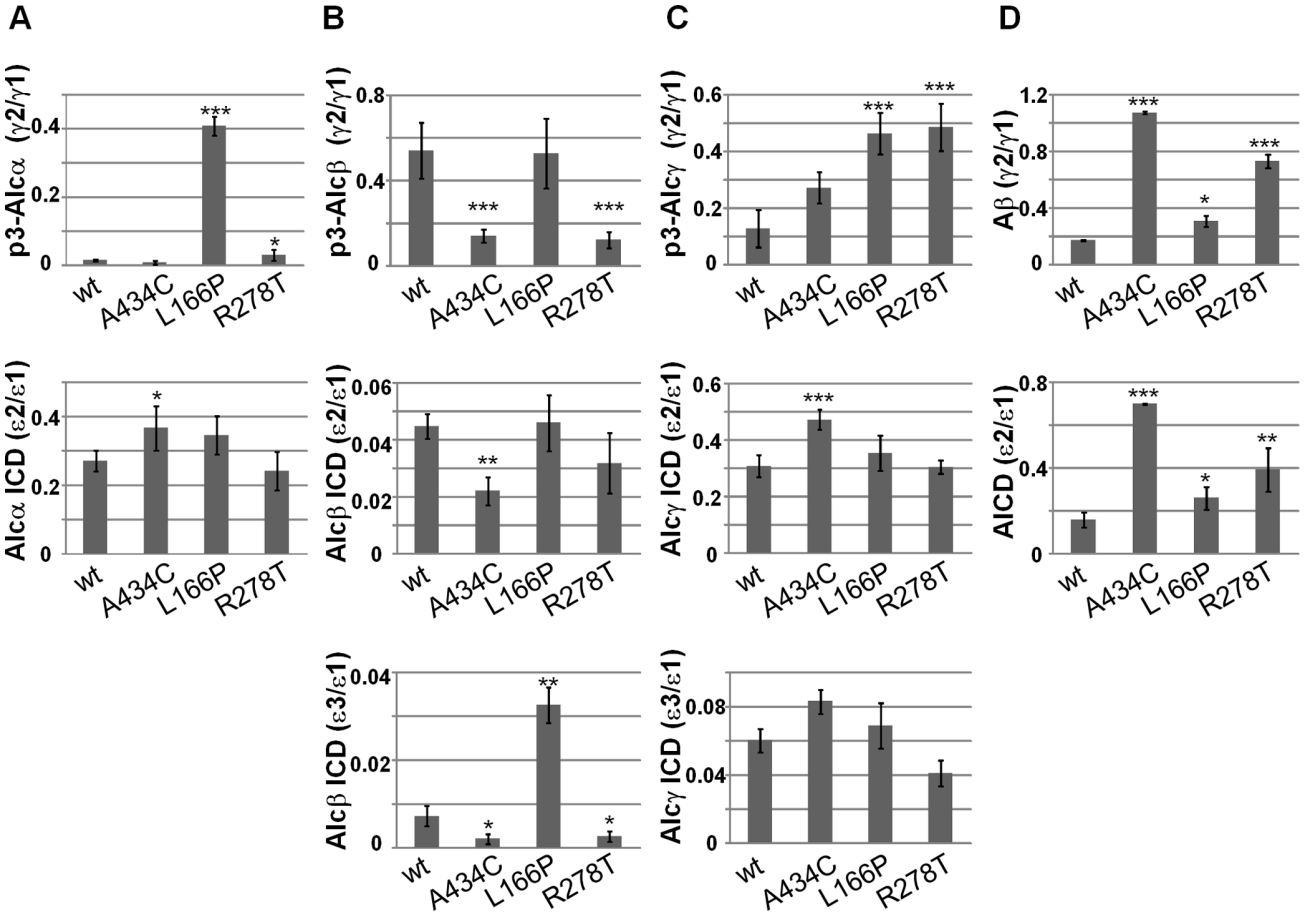
Magnitudes of the minor to major ratios of γ-cleavage and ε-cleavage in Alcs and APP from cells expressing FAD-linked PS1 mutants. Minor to major p3-Alc ratio (γ2/γ1, first rows) was determined by quantitative MS analysis of p3-Alc secreted by HEK293 cells expressing Alc-ΔC-FLAG with either wild-type PS1 or PS1 carrying a FAD-linked mutation (A434C, L166P, or R278T) (**Fig. S5**). Minor to major Alc ICD ratios (ε2/ε1, second rows; ε3/ε1, third rows) were also determined by MS analysis of Alc ICD-ΔC-FLAG generated by *in vitro* γ-secretase assay with membranes from the same cells (**Fig. S5**). **A. Comparison of Alcα γ-cleavage ratio to ε-cleavage ratio.** The ratio (γ2/γ1) of p3-Alcα2N+38 (minor) to p3-Alcα2N+35(major) (first row) and the ratio (ε2/ε1) of the Alcα ICD-ΔC-FLAG product cleaved at ε2 (minor) to the product cleaved at ε1 (major) (second row) are shown. **B. Comparison of Alcβ γ-cleavage ratio to ε-cleavage ratio.** The ratio (γ2/γ1) of p3-Alcβ37 (minor) to p3-Alcβ40 (major) (first row) and the ratio (ε2/ε1 or ε3/ε1) of the Alcβ ICD-ΔC-FLAG product cleaved at ε2 or ε3 (minor) to the product cleaved at ε1 (major) (second row, ε2/ε1; third row, ε3/ε1) are shown. **C. Comparison of Alcγ γ-cleavage ratio to ε-cleavage ratio.** The ratio (γ2/γ1) of p3-Alcγ34 (minor) to p3-Alcγ31 (major) (first row) and the ratio (ε2/ε1 or ε3/ε1) of the Alcγ ICD-ΔC-FLAG product cleaved at ε2 or ε3 (minor) to the product cleaved at ε1 (major) (second row, ε2/ε1; third row, ε3/ε1) are shown. **D. Comparison of APP γ-cleavage ratio to ε-cleavage ratio.** The ratio (γ2/γ1) of Aβ42 (minor) to Aβ40 (major) (first row) and the minor to major ratio of AICD (second row, ε2/ε1) are shown. Net Aβ values are shown in **Table S2**, and representative mass spectra of AICD-FLAG are shown in **Fig. S5D**. Statistical analysis was performed using Dunnett’s multiple comparison test. Significance is indicated relative to the ratio of wild-type PS1 (wt) (mean ± S.E., n = 4, *P<0.05, **P<0.001, ***P<0.0001).

Previous observation revealed that FAD-linked PS1 mutations altered the ratio of γ-cleavages of Alcα, Alcβ and Alcγ, as observed in APP, although the magnitudes of altered γ-cleavage varied across these substrates [17]. We next examined whether the altered γ-cleavage of Alcs in cells expressing a FAD-linked PS1 mutation relates to the alteration of first ε-cleavage position. Alcs-ΔC-FLAG proteins were coexpressed in cells together with wild-type PS1 or FAD-linked PS1 mutants. The p3-Alcs secreted by cells were analyzed with MALDI-TOF/MS (**Fig. S5, upper rows in panels A to C**), and the minor/major ratios of γ-cleavage products were determined (**Fig. 2A to C** **, upper panels**). Alcs ICD-FLAG was generated by *in vitro* γ-secretase assay with membrane and identified with MALDI-TOF/MS (**Fig. S5, lower rows in panels A to C**), and the minor/major ratios of ε-cleavages were compared to those of γ-cleavages (**Fig. 2A to C** **, middle and lower panels**). APP CTFβ/C99-FLAG was also analyzed for major (Aβ40) and minor (Aβ42) γ-cleavage products by sandwich ELISA (sELISA; **Table S2**) along with major and minor ε-cleavage products, APP intracellular domain fragment (AICD-FLAG; **Fig. S5D**). The minor/major ratio of γ-cleavage was compared to that of ε-cleavage (**Fig. 2D**). Positions of APP γ- and ε-cleavage sites are indicated for comparison with the positions in Alcs (**Fig. 1C**).

In APP, as expected, the magnitudes of altered ε-cleavage demonstrated minor/major (ε2/ε1) ratios well correlated with the altered γ-cleavage minor/major (Aβ42/Aβ40) ratios in cells expressing the wild type and FAD-linked mutants of PS1 (R^2^ = 0.7356; **Fig. S6D and** **Fig. 2D**). In contrast to APP, Alcs tended to show that the first-determined ε-cleavage position is not necessarily prerequisite to determine a specific γ-cleavage position (compare upper panels with middle and lower panels in **Fig. 2A–C**). In Alcα, minor γ-cleavage to generate p3-Alcα38 increased remarkably in cells expressing PS1 L166P and less significantly in cells expressing PS1 R278T, while no significant effect was observed in cells expressing PS1 A434C mutant, when compared to cells expressing wild-type PS1. Minor ε-cleavage (ε2) increased slightly in cells expressing PS1 A434C mutant, while no significant alternation was detected in cells expressing PS1 L166P or R278T mutants when compared to cells expressing wild-type PS1 (**Fig. S5A**). The comparison of the minor/major (38/35) ratio of γ-cleavage with minor/major (ε2/ε1) ratio of ε-cleavage in cells expressing the respective PS1 mutants suggests that covariance between the magnitude of ε-cleavage and γ-cleavage positions alteration in Alcα was low (R^2^ = 0.1597; **Fig. S6A and** **Fig. 2A**).

We performed identical analyses for Alcβ and Alcγ (**Fig. 2B and C**). In Alcβ, the minor/major ratio of γ-cleavage is indicated as the p3-Alcβ37/p3-Alcβ40 (γ2/γ1) ratio. Because Alcβ demonstrated at least three ε-cleavage sites, a major ε1 site with minor ε2 and ε3 sites (**Fig. 1B**), we examined both ε2/ε1 and ε3/ε1 ratios to determine the minor/major ratio of ε-cleavage. Minor γ-cleavage was significantly reduced in cells expressing PS1 A434C and R278T mutants (**Fig. S5B**). Decreased minor ε-cleavage seemed to occur in cells expressing PS1 A434C, as reflected in both the ε2/ε1 and ε3/ε1 ratios (**Fig. 2B**). PS1 R278T decreased the ε3/ε1 ratio significantly and tended to decrease the ε2/ε1 ratio, but not significantly. PS1 L166P did not affect the ε2/ε1 ratio, but the ε3/ε1 ratio was greatly increased. These results may suggest that alteration of ε-cleavage tends to reflect the position of the γ-cleavage site in Alcβ (R^2^ = 0.8518 for ε2/ε1 ratio versus γ2/γ1 ratio and R^2^ = 0.4675 for ε3/ε1 ratio versus γ2/γ1; **Fig. S6B**). However, upon careful analysis of **Fig. 2B**, the ratios of ε2/ε1 and ε3/ε1 are very low (<0.05), while the ratio of γ2/γ1 is approximately 0.5 in wild-type PS1 and L166P mutant, and 0.15 in A434C and R278T mutants. This observation indicates that both γ1 and γ2 cleavages are largely derived from ε1 cleavage position in Alcβ, or in other words, the ε1 site is exclusively dominant among Alcβ ε-cleavage sites. Therefore, one dominant ε-site is likely to determine two γ-sites, and the alteration of γ-site is not affected by a small magnitude of alternation at the ε-site position in Alcβ.

In Alcγ, the minor/major ratio of γ-cleavage is indicated as the p3-Alcγ34/p3-Alcγ31 (γ2/γ1) ratio. Alcγ demonstrated three ε-cleavage sites, a major ε1 site with minor ε2 and ε3 sites (**Fig. 1C**). Thus, as in the case of Alcβ, we examined both ε2/ε1 and ε3/ε1 ratios to determine the minor/major ratio of ε-cleavage. Cells expressing FAD-linked PS1 L166P and R278T mutants demonstrated significantly increased minor γ-cleavage, while only the PS1 A434C mutant demonstrated increased ε2/ε1 ratio, and no PS1 mutants demonstrated a significant change in ε3/ε1 ratio compared to wild-type PS1 (**Fig. 2C** **and Fig. S5C**). These results also suggest that alteration of ε-cleavage positions does not largely correlate with the alteration of Alcγ γ-cleavage sites (R^2^ = 0.0201 for ε2/ε1 ratio versus γ2/γ1 ratio and R^2^ = 0.1372 for ε3/ε1 ratio versus γ2/γ1 ratio; **Fig. S6C and** **Fig. 2C**).

Overall, these findings indicate that alteration of ε-cleavage sites in Alcs does not influence the determination of γ-cleavage site, unlike APP, which demonstrates a significant covariance of changes in magnitude between ε- and γ-cleavage products.

### A specific ε-cleavage Site is not Necessarily Prerequisite to Determine a Specific γ-cleavage Positions in Alcs

**Figure 3:**
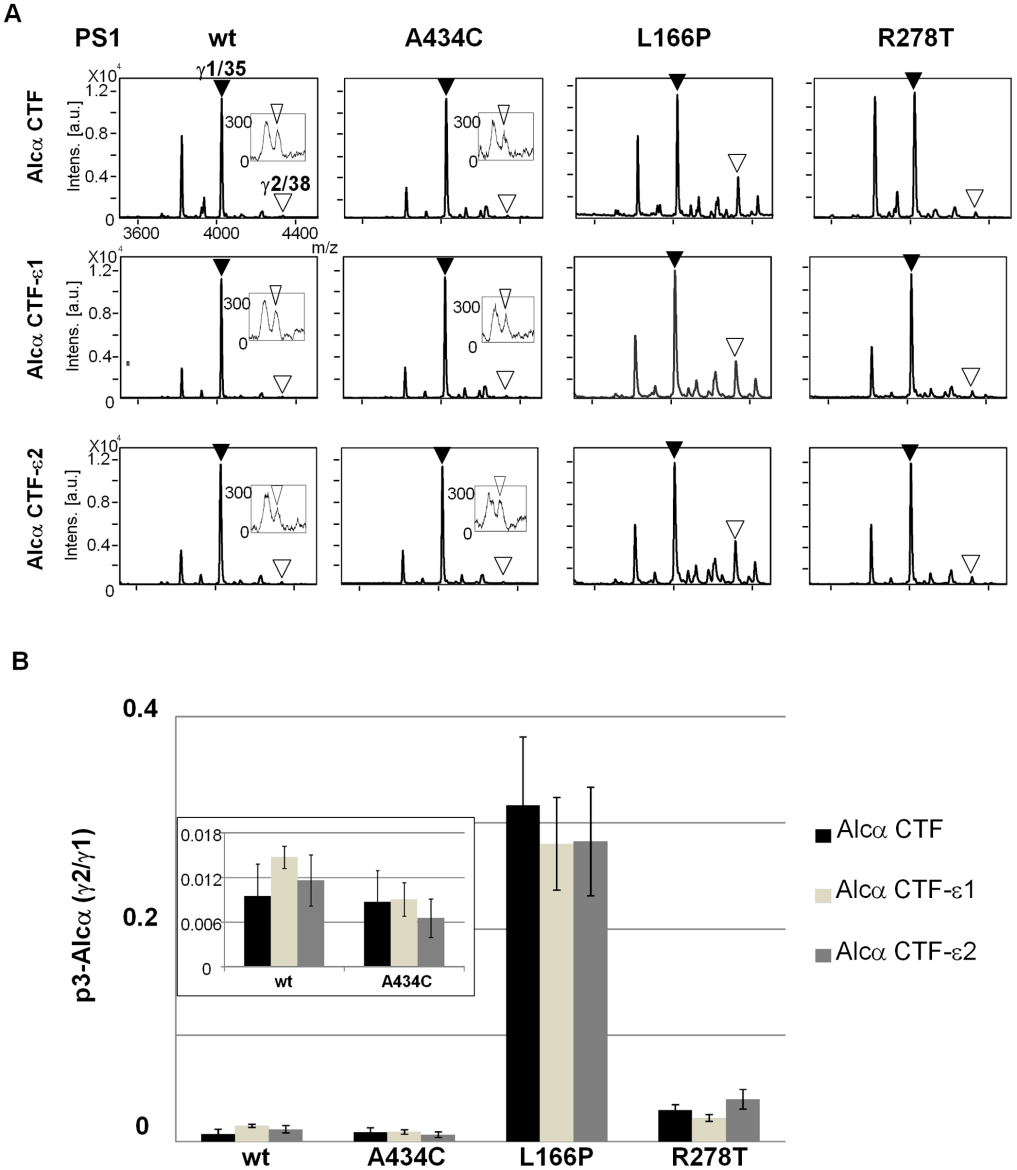
Alteration of ε-cleavage is not necessarily prerequisite to determine a specific γ-cleavage site in Alcα. A**.** Representative MS spectra of p3-Alcα secreted by HEK293 cells expressing Alcα CTF, Alcα CTF-ε1, or Alcα CTF-ε2 with either wild-type PS1 (wt) or a FAD-linked PS1 mutant (A434C, L166P, or R278T). The p3-Alcα species in cell culture media were immunoprecipitated and subjected to MALDI-TOF/MS analysis. Closed arrowheads indicate the major product with γ1 site (p3-Alcα2N+35, “γ1/35”), while open arrowheads indicate the minor product with γ2 site (p3-Alcα2N+38, “γ2/38”). The spectra of the minor p3-Alcα38 product are enlarged in windows in which intensity of 300 on the y-axis corresponds to 0.03 in the original panels. **B.** The peak area of p3-Alcα2N+38 (minor species) was compared with that of p3-Alcα2N+35 (major species), and the minor to major ratios (p3-Alcα2N+38/p3-Alcα2N+35) are indicated asγ2/γ1. Statistical analysis was performed using one-way analysis of variance followed by the Tukey-Kramer multiple comparison test (means ± S.E., n = 4). Significance in comparison to the ratio of Alcα CTF was not observed in cells expressing wild-type PS1 (wt) or FAD-linked PS1 mutants. The columns of “wt” and “A434C” are enlarged in window.

**Figure 4:**
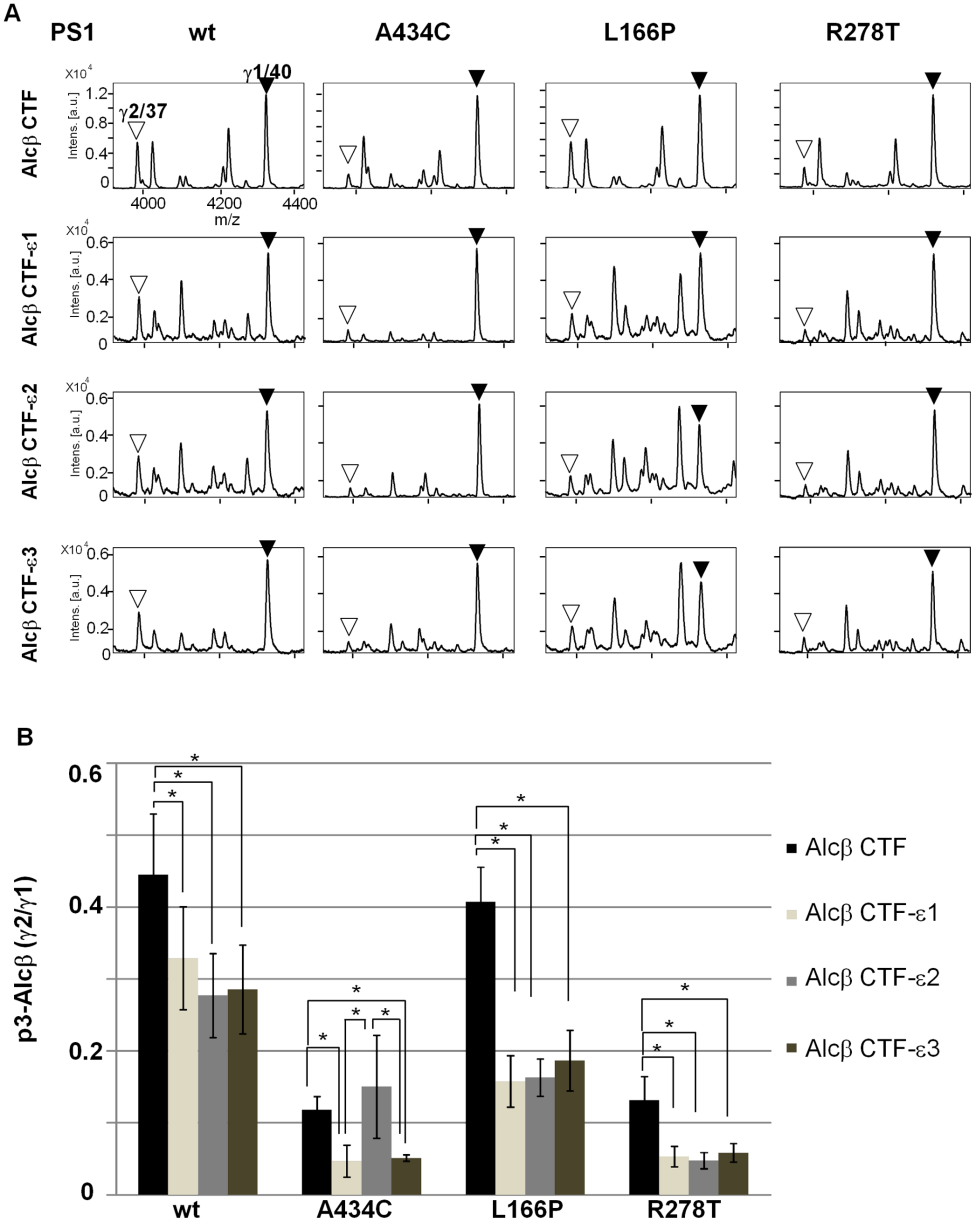
Alteration of ε-cleavage is not necessarily prerequisite to determine a specific γ-cleavage site in Alcβ. A. Representative MS spectra of p3-Alcβ species secreted by HEK293 cells expressing Alcβ CTF, Alcβ CTF-ε1, Alcβ CTF-ε2, or Alcβ CTF-ε3 with either wild-type PS1 (wt) or a FAD-linked PS1 mutant (A434C, L166P, or R278T). The p3-Alcβ species in cell culture media were immunoprecipitated and subjected to MALDI-TOF/MS analysis. Closed arrowheads indicate the major product with γ1 site (p3-Alcβ40, “γ1/40”), while open arrowheads indicate the minor product with γ2 site (p3-Alcβ37, “γ2/37”). **B.** The peak area of p3-Alcβ37 (minor species) was compared with that of p3-Alcβ40 (major species), and the minor to major ratios (p3-Alcβ37/p3-Alcβ40) are indicated as γ2/γ1. Statistical analysis was performed using one-way analysis of variance followed by the Tukey-Kramer multiple comparison test (mean ± S.E., n = 4, *P<0.05).

**Figure 5:**
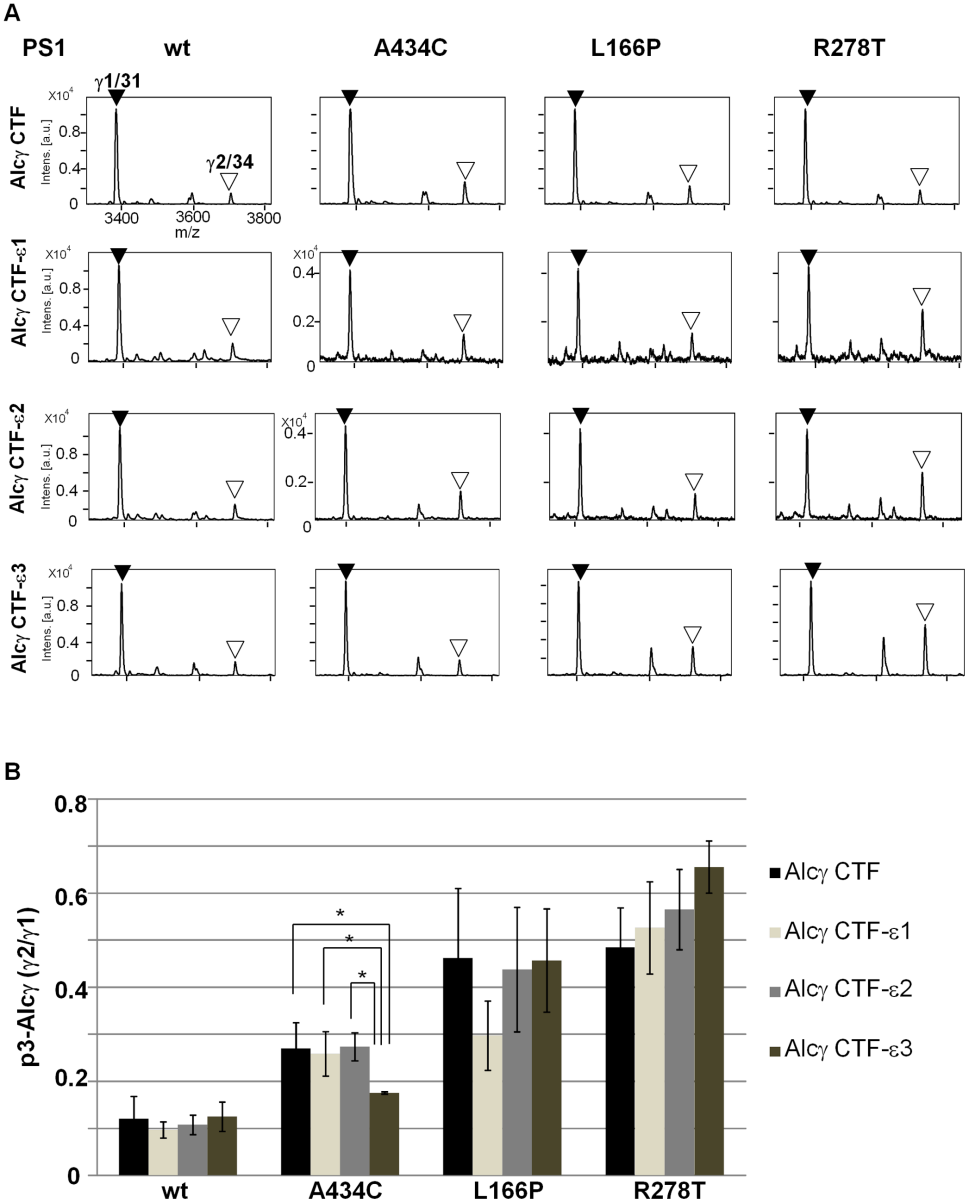
Alteration of ε-cleavage is not necessarily prerequisite to determine a specific γ-cleavage site in Alcγ. A. Representative MS spectra of p3-Alcγ secreted by HEK293 cells expressing Alcγ CTF, Alcγ CTF-ε1, Alcγ CTF-ε2, or Alcγ CTF-ε3 with either wild-type PS1 (wt) or FAD-linked PS1 mutants (A434C, L166P, R278T). The p3-Alcγ species in cell culture media were immunoprecipitated and subjected to MALDI-TOF/MS analysis. Closed arrowheads indicate the major product with γ1 site (p3-Alcγ31, “γ1/31”), while open arrowheads indicate the minor product with γ2 site (p3-Alcγ34, “γ2/34”). **B.** The peak area of p3-Alcγ34 (minor species) was compared with that of p3-Alcγ31 (major species), and the ratios (p3-Alcγ34/p3-Alcγ31) are indicated as γ2/γ1. Statistical analysis was performed using one-way analysis of variance followed by the Tukey-Kramer multiple comparison test (mean ± S.E., n = 4, *P<0.05).

To further examine whether one ε-cleavage position can determine a specific γ-cleavage position, we expressed Alcs truncated at ε-cleavage sites in cells expressing wild-type and FAD-linked mutants of PS1, and analyzed the alteration of γ-cleavage sites (**Fig. 3** **** **Fig. 4** **** **Fig. 5**). Alcα CTF-ε1 (truncated at site ε1) and Alcα CTF-ε2 (truncated at site ε2), along with Alcα CTF, were expressed in cells **(** **Fig. 3** **and Fig. S7A**). The p3-Alcα in the culture media was analyzed with MALDI-TOF/MS (**Fig. 3A**), and the minor/major (p3-Alcα 2N+38/p3-Alcα 2N+35 or γ2/γ1) ratios were determined (**Fig. 3B**). Production levels of p3-Alcα were highly similar between Alc CTF and Alc CTF-ε. The minor/major (γ2/γ1) ratios of p3-Alcα from cells expressing wild-type (wt) and FAD-linked mutants of PS1 (A434C, L166P, and R278T) did not significantly differ between Alcα CTF and Alcα CTF-ε. These results indicate that both ε1 and ε2 cleavage generate ratios of the major γ-cleavage product that are identical to those of Alcα CTF with an intact cytoplasmic region. This analysis clearly indicates that the first-determined ε-cleavage is not necessarily prerequisite to determine γ-cleavage position; that is, both ε1 and ε2-sites predominantly reach the γ1 site as the major γ-cleavage position. We confirmed that FAD-linked PS1 mutation L166P demonstrated the greatest effect on increasing the generation of minor γ2-cleaved product [**13**] (**Fig. S5A**), but the effect of this mutation may be not due to the position of initial ε-cleavage site.

The same analysis was performed for Alcβ (**Fig. 4** **and Fig. S7B**) and Alcγ (**Fig. 5** **and Fig. S7C**). Alcβ CTF-ε1, Alcβ CTF-ε2, Alcβ CTF-ε3, and Alcβ CTF were expressed in cells along with different forms of PS1, and the secreted p3-Alcβ was analyzed (**Fig. 4A**). Alcβ CTF truncated at ε1, ε2, orε3 generated almost identical levels of major γ1 (p3-Alcβ40) and minor γ2 (p3-Alcβ37) products, but their production levels decreased to 50–60% of those of Alcβ CTF including an intact cytoplasmic region (note “*Intens*” shown in **Fig. 4A**). The γ2/γ1 ratios of the three truncated Alcβ CTF-ε species were not largely affected by the FAD-linked PS1 mutation, except for the A434C mutant, in which Alcβ CTF-ε1 and Alcβ CTF-ε3 decreased the γ2/γ1 ratio.

The results largely demonstrated identical minor/major (γ2/γ1) ratios for γ-cleavage products among AlcβCTF-ε species truncated at the ε1, ε2, and ε3 sites **(** **Fig. 4B**). However, Alc CTFβ-ε significantly decreased the minor γ2-cleaved product (p3-Alcβ37), resulting in the decrease of the minor/major (γ2/γ1) ratio compared to that of Alcβ CTF, which seems to differ slightly from the case of Alcα and Alcγ (compare **Fig. 4B** with **Figs. 3B** **and** **5B**). However, overall, the results indicate that one ε-cleavage position has no determining effect on the dominance of a specific γ-cleavage position, and that the effect of FAD-linked PS1 mutations on the ratio of γ-cleaved products is not largely due to the alteration of ε-cleavage position.

Alcγ CTF-ε truncated at ε1, ε2, and ε3 sites also secreted p3-Alcγ with a fixed minor/major (p3-Alcγ34/p3-Alcγ31 or γ2/γ1) ratio that was highly similar to the ratio of Alcγ CTF, with the exception of Alcγ CTF-ε3 in cells expressing A434C PS1 mutant (**Fig. 5** **and Fig. S7C**). The production levels of p3-Alcγ derived from Alcγ CTF-ε positions were highly similar overall to that of p3-Alcγ derived from Alcγ CTF with the entire C-terminal in cells expressing wild-type PS1, but decreased by 40% for Alcγ CTF-ε1 and Alcγ CTF-ε2 in cells expressing FAD-linked PS1 mutants (note “*Intens*” shown in **Fig. 5A**). This analysis indicates that the alteration of ε-cleavage position does not affect the determination of γ-cleavage position of Alcγ.

Taken together, these findings indicate that the position of physiological ε-site is not necessarily prerequisite to determine a specific γ-cleavage position in Alcs. Moreover, the alteration of initial ε-cleavage site does not contribute to the changes of their minor/major ratio of γ-cleaved products in cells expressing FAD-linked mutation of PS1. The observation with Alcs may differ from the conclusion obtained from APP CTFs truncated at ε-sites. In APP, cells express CTF 1–49 (ε1 site) secreted predominantly Aβ40 while those expressing CTF 1–48 (ε2 site) secreted preferentially Aβ42 [**8**].

### Alteration of γ-cleavage upon Disturbance of Physiological ε-cleavage Sites in Alcs

**Figure 6:**
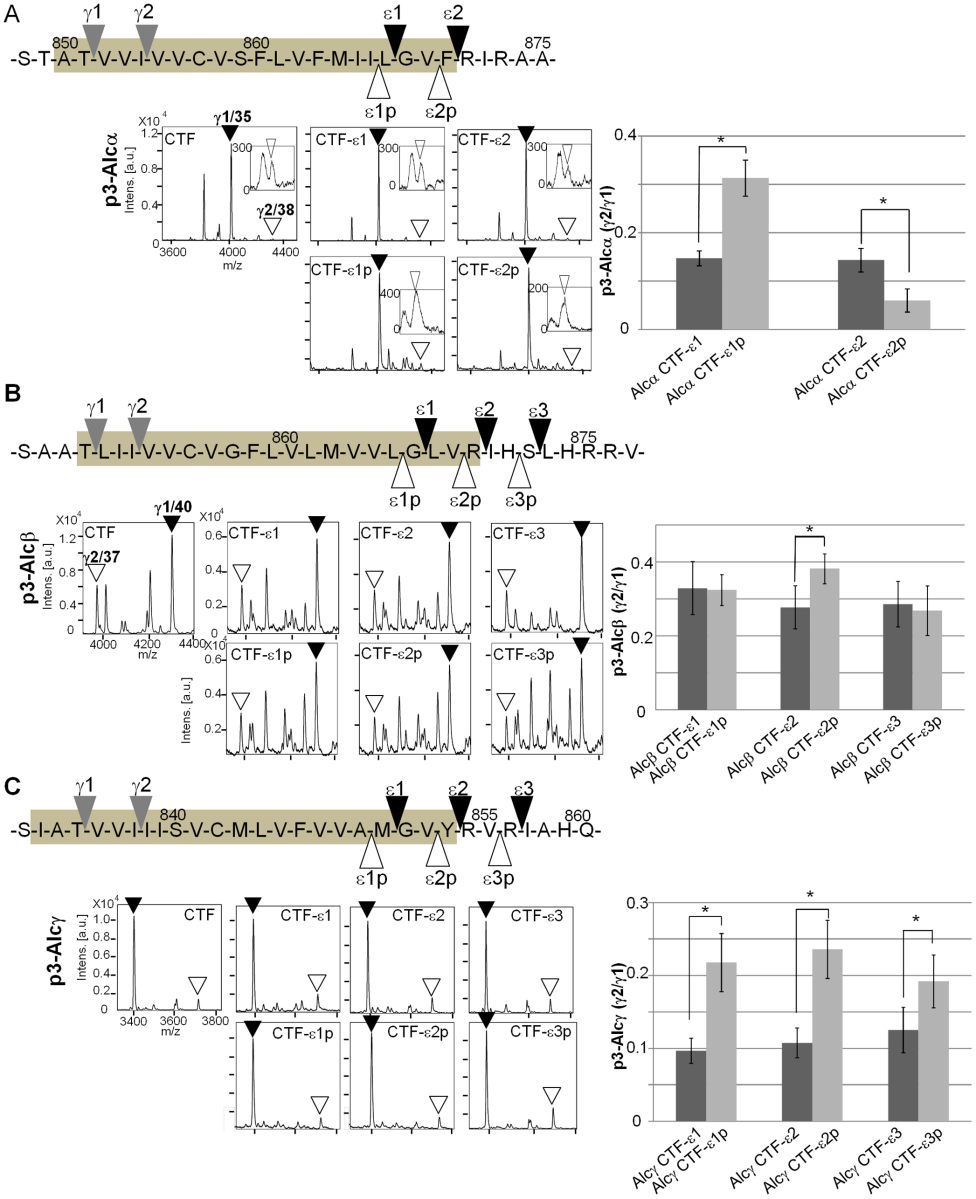
Alteration of Alcs γ-cleavage when physiological ε-cleavage sites are replaced with non-physiological/pseudo-ε-cleavage sites. A. Positions of the physiological major and minor (ε1 and ε2) and pseudo- (ε1p and ε2p) ε-cleavage sites (upper left) are shown along with the physiological major and minor γ-cleavage sites (γ1 and γ2). The shaded amino acid sequence indicates a putative membrane-embedded region. Non-physiological/pseudo-ε-cleavage sites were designed by shifting one residue toward the N terminal of the physiological ε-cleavage sites. Representative MS spectra of p3-Alcα secreted by HEK293 cells expressing Alcα CTF, Alcα CTF-ε1, Alcα CTF-ε2, Alcα CTF-ε1p, or Alcα CTF-ε2p are shown (lower left panels). The major species p3-Alcα2N+35 with γ1 site (γ1/35, closed arrowheads) and minor species p3-Alcα2N+38 with γ2 site (γ2/38, open arrowheads) are indicated. The peak area of p3-Alcα2N+38 was compared with that of p3-Alcα2N+35, and the ratios (p3-Alcα2N+38/p3-Alcα2N+35) are indicated as γ2/γ1 (right panel). The spectra of minor species p3-Alcα38 are enlarged in windows in which intensities of 200, 300, and 400 on the y-axis correspond to 0.02, 0.03 and 0.04 in the original panels. **B.** Positions of the physiological major and minor (ε1, ε2, and ε3) and pseudo- (ε1p, ε2p, and ε3p) ε-cleavage sites (upper left) are shown along with the physiological major and minor γ-cleavage sites (γ1 and γ2). Representative MS spectra of p3-Alcβ secreted by HEK293 cells expressing Alcβ CTF, Alcβ CTF-ε1, Alcβ CTF-ε2, Alcβ CTF-ε3, Alcβ CTF-ε1p, Alcβ CTF-ε2p, or Alcβ CTF-ε3p are shown (lower left). The major species p3-Alcβ40 withγ1 site (γ1/40, closed arrowheads) and minor species p3-Alcβ37 with γ2 site (γ2/37, open arrowheads) are indicated. The peak area of p3-Alcβ37 was compared with that of p3-Alcβ40, and the ratios (p3-Alcβ37/p3-Alcβ40) are indicated asγ2/γ1 (right panel). **C.** Positions of the physiological major and minor (ε1, ε2, and ε3) and pseudo- (ε1p, ε2p, and ε3p) ε-cleavage sites (upper left) are shown along with the physiological major and minor γ-cleavage sites (γ1 and γ2). Representative MS spectra of p3-Alcγ secreted by HEK293 cells expressing Alcγ CTF, Alcγ CTF-ε1, Alcγ CTF-ε2, Alcγ CTF-ε3, Alcγ CTF-ε1p, Alcγ CTF-ε2p, or Alcγ CTF-ε3p are shown (lower left). The major p3-Alcγ31 with γ1 site (γ1/31, closed arrowhead) and minor p3-Alcγ34 with γ2 site (γ2/34, open arrowhead) are indicated. The peak area of p3-Alcγ34 was compared with that of p3-Alcγ31, and the ratios (p3-Alcγ34/p3-Alcγ31) are indicated as γ2/γ1 (right panel). (**A–C**) The ratios of products from the pseudo-site were compared to those from the respective physiological sites. Statistical analysis was performed by Student's t test (mean ± S.E., n = 4, *P<0.05).

We next asked whether the γ-cleavage sites are altered when Alc CTFs are initially truncated at artificial ε-cleavage positions (**Fig. 6**). Pseudo-ε-cleavage sites were designated toward the N termini of the physiological major and minor ε-cleavage sites. Alcα CTFs with physiological ε-cleavage sites, Alcα CTF-ε1 and Alcα CTF-ε2, or Alcα CTFs with artificial/pseudo ε-cleavage sites, Alcα CTF-ε1p (ε1 pseudo) and Alcα CTF-ε2p (ε2 pseudo), were expressed in cells along with Alcα CTF. The p3-Alcα in media was analyzed with MALDI-TOF/MS to determine the minor/major (p3-Alcα38/p3-Alcα35 or γ2/γ1) ratio. Production levels of p3-Alcα did not largely change between Alcα CTF-ε and Alcα CTF-εp. Surprisingly, Alcα CTF-ε1p significantly increased the minor/major ratio of γ-cleaved products, and Alcα CTF-ε2p remarkably decreased the minor/major ratio (**Fig. 6A**), suggesting that alternation of γ-cleavage position may be affected by movement of the ε-cleavage position by one amino acid.

We confirmed this phenomenon with Alcβ CTF and Alcγ CTF truncated with pseudo-ε-sites (**Fig. 6B and C**). Production levels of p3-Alcβ derived from Alcβ CTF-ε positions were ∼60% of those of Alcβ CTF (note “*Intens*” shown in **Fig. 6B**), but no significant differences in minor/major (γ2/γ1) ratio were observed between the Alcβ CTF-ε and Alcβ CTF-εp. Only Alcβ CTF-ε2p demonstrated a small but significant increase in the minor/major ratio of γ-cleavage. Production levels of p3-Alcγ derived from Alcγ CTF-ε positions were unchanged across all Alcγ CTFs with physiological and pseudo-ε-sites. All three pseudo-ε-sites of Alcγ CTF significantly increased the minor/major (γ2/γ1) ratio of γ-cleavage products. In Alcβ CTF, several MS signals were detected along with the γ1 and γ2 signals (**Fig. 6B**). These signals do not reflect products derived from Alcβ CTFs because cells without expression of Alcβ CTFs also generated these signals (**Fig. S8**).

Overall, in Alcs or at least in Alcα and Alcγ, movement of the physiological ε-cleavage position by one amino acid may be a possible mechanism to induce the alteration of γ-cleavage site dominance, although the changes to cellular condition that are able to induce the movement from the physiological sites of ε-cleavage remain unknown. Importantly, no additional γ-cleavage sites were generated from the artificial/non-physiological positions of ε-cleavage used for all Alcs. Thus, again, the initial ε-cleavage site is not necessarily prerequisite to determine the position of γ-cleavage.

## Discussion

In a previous study, the magnitude of Alcs γ-cleavage alteration in cells expressing FAD-linked PS1 mutants varied and differed from APP [17], suggesting that the determination of initial intramembrane ε-cleavage may not be necessarily prerequisite to cleave at a specific γ-cleavage site in the case of Alcs. We determined two to three ε-cleavage sites each in Alcα, Alcβ and Alcγ, as observed in APP and Notch [20]–[24]. One represented the major ε-site at which Alc CTF was predominantly cleaved. We found that some FAD-linked PS1 mutations affected the ratio of minor to major ε-cleavage, but this alteration to the ratio of minor to major γ-cleavage was not always apparent. Similarly, some FAD-linked PS1 mutations did not remarkably influence in the selection of ε-site but significantly affected the γ-cleavage site. These properties of Alcs intramembrane cleavage by γ-secretase differ from those of APP, in which changes to the ratio of minor to major γ-cleavage were consistent with changes to the ratio of minor to major ε-cleavage [10], [12], [25]. Therefore, we propose that the mechanism of intramembrane cleavage by γ-secretase is not regulated identically between Alcs and APP. Such relationship between the γ-site position and the ε-cleavage site in Alcs was demonstrated with several different types of experiment, and our current findings suggest that the endophenotype of γ-secretase malfunction appears to affect either γ-cleavage or ε-cleavage position in Alcs.

Notably, Alcs CTF possessing artificial/non-physiological pseudo-ε-sites at their C-termini did not generate novel γ-sites, while the ratio of minor to major γ-cleavage products changed significantly in Alcα and Alcγ, but less so in Alcβ. This observation suggests that the selection of ε-site may be relatively flexible, but the position of the γ-site is rigid, and that the minor to major ratio of the γ-cleavage site rather than the positional alteration of ε-cleavage or the minor to major ratio of ε-cleavage site is more likely to reflect the endophenotype of γ-secretase malfunction. This may be consistent with a recent report that γ-secretase malfunction tends to generate Aβ42 instead of Aβ38 which should be generated finally from APP by an entire γ-secretase [14], indicating a selection of different γ-site positions as an endophenotype of γ-secretase malfunction. Furthermore, our study indicates that the shift of physiological ε-cleavage to an unusual ε-site can alter the minor/major ratio of γ-site cleavage. It remains for future studies to determine whether such non-physiological ε-cleavage of Alcs occurs in cells.

The present findings may be an important step to revealing the mechanism of γ-secretase malfunction in SAD, which differs from that in FAD. Because altered γ-secretase processing was observed as an endophenotype of p3-Alcα in CSF of some SAD subjects [18], Alcs may be more sensitive substrates to detect γ-secretase malfunction [26].

## Materials and Methods

### Plasmid Construction and Stable Cell Lines Expressing PS1

The human Alcadein cDNAs, hAlcα1, hAlcβ, and hAlcγ and plasmids encoding human PS1 cDNAs have been described [17]. FAD-linked mutations were introduced by PCR-based site-directed mutagenesis to generate pcDNA3.1-PS1L166P, pcDNA4-PS1R278T, and pcDNA4-PS1A434C. HEK293 cells were transfected with these plasmids, and cells stably expressing PS1 were cloned as described [17].

### Antibodies

Rabbit polyclonal anti-Alcα antibody UT135 was raised against a peptide composed of Cys plus the sequence between positions 839 and 851 (NPHPFAVVPSTAT+C) of human Alcα. Rabbit polyclonal anti-Alcβ antibody UT143 was raised against a GST-fusion protein containing the sequence between positions 819 and 847 (FLHRGHQPPPEMAGHSLASSHRNSMIPSA) of human Alcβ. Rabbit polyclonal anti-Alcγ antibody UT166 was raised against a peptide composed of Cys plus the sequence between positions 823 and 834 (C+IQHSSVVPSIAT) of human Alcγ. These Alc-specific antibodies were specific to their respective p3-Alc targets with the exception of UT166, which exhibited cross-reactivity to p3-Alcα (data not shown). These antibodies were used to isolate and detect p3-Alc [17]. The monoclonal anti-FLAG antibody (M2) was purchased from Sigma-Aldrich.

### MALDI-TOF/MS and -MS/MS Analysis of p3-Alc Secreted into the Culture Medium

HEK293 cells (8–9×10^6^) were transfected with plasmids (6 µg) in Lipofectamine 2000 according to the manufacturer's protocol (Invitrogen) for 24 h. The p3-Alcα, p3-Alcβ, and p3-Alcγ that were secreted into the medium (10 ml) were recovered by immunoprecipitation in the presence of protease inhibitor cocktail (5 µg/ml chymostatin, 5 µg/ml leupeptin, and 5 µg/ml pepstatin) as described [17] using the polyclonal anti-p3-Alcα UT135 (4 µg of affinity purified IgG), polyclonal anti-p3-Alcβ UT143 (100 µl of serum), and polyclonal anti-p3-Alcγ UT166 (100 µl of serum) antibodies, respectively, and Protein G-Sepharose beads. The beads were sequentially washed with Wash buffer I (10 mM Tris-HCl (pH 8.0), 140 mM NaCl, 0.1% (w/v) *n*-octyl-D-glucoside, 0.025% (w/v) sodium azide) and Wash buffer II (10 mM Tris-HCl (pH 8.0), 0.025% (w/v) sodium azide), and samples were then eluted with a solution of trifluoroacetic acid/acetonitrile/water (1∶20:20) saturated with sinapinic acid. The samples were dried on a target plate, and MALDI-TOF/MS analysis was performed using an UltraflexII TOF/TOF (Bruker Daltonics, Bremen, Germany). Molecular masses were calibrated using the peptide calibration standard (Bruker Daltonics) [17], [18]. The quantitative accuracy of mass spectrometric analysis with immunoprecipitation was confirmed previously [17], and molecular masses of p3-Alc species measured with MALDI-TOF/MS were compared with theoretical values to confirm the accuracy of mass spectrometric analysis (**Table S1**). Furthermore, we confirmed that the quantity of peptides is not affected by coexistence with the increased amount of other peptides, suggesting specific ion suppression of peptide does not occur in this assay (**Figs. S9 and S10**).

### Membrane Incubation for Substrate Cleavage by γ-secretase (in vitro γ-secretase Assay)

To detect Alc ICD, HEK293 cells stably expressing wild-type PS1 or PS1 with a FAD-linked mutation were transfected with plasmid (6 µg) in Lipofectamine 2000 according to the manufacturer's protocol (Invitrogen). After 20-h culture of cells, the γ-secretase inhibitor DAPT (10 µM, 3,5-(Difuorophenyl)acetyl-L-alanyl-L-2-phenylglycine *t*-butyl ester) was added to the medium, and cells were cultured for an additional 4 h. The cells were then harvested and lysed in 500 µl of homogenizing buffer (20 mM HEPES, 150 mM NaCl, 10% glycerol, 5 mM EDTA, 5 mM EGTA) by passing through a 27-gauge needle 30 times on ice. After the removal of unbroken organelles and nuclei by centrifugation at 3,000 rpm for 10 min at 4°C, the membranes were precipitated by centrifugation at 100,000× *g* for 60 min at 4°C. The crude membrane fraction was washed once with homogenizing buffer and re-suspended in an assay buffer (20 mM HEPES, 150 mM NaCl, 10% glycerol, 5 mM EDTA, 5 mM EGTA, 10 µM amastatin, 0.1 µM arphamenine A). After incubation for 2 h at 37°C for substrate cleavage by γ-secretase, the membrane suspension was subjected to centrifugation at 100,000× g for 30 min at 4°C. The supernatant including the ε-site-cleaved product with FLAG-tag was subjected to immunoprecipitation with anti-FLAG antibody and Protein G-Sepharose beads. Immunoreactive proteins were analyzed by MALDI-TOF/MS. Molecular masses of p3-Alc and Alc ICD species generated by *in vitro* γ-secretase assay and measured with MALDI-TOF/MS were compared with theoretical values to confirm the accuracy of mass spectrometric analysis (**Table S1**).

### Quantitative Aβ Assay

Aβ40 and Aβ42 secretion into the medium were quantified by sELISA as described [27], and their net values are shown in **Table S2**.

## Supporting Information

Figure S1 **Schematic structure of Alc-ΔC-FLAG fusion proteins used for the** ***in vitro*** **γ-secretase assay to determine** ε**-cleavage sites.** Cytoplasmic regions of Alcα, Alcβ and Alcγ were truncated at the indicated positions and fused to FLAG-tag sequence. Amino acid numbering corresponds to human Alcadein α1 (971 amino acids), Alcadein β (956 mino acids), and Alcadein γ (956 amino acids) [16]. Primary α-cleavage sites are indicated with open arrowheads. CTF (*light gray shading on Alc proteins*), C-terminal region of Alc cleaved by α-secretase.
(TIF)

Figure S2 **Representative MS spectra of Alc ICD-ΔC-FLAG generated by** ***in vitro*** **γ-secretase assay with membranes derived from cells expressing wild-type PS1 or the dominant-negative PS1 mutant D385A.** Membranes from cells expressing Alcα-ΔC-FLAG (left), Alcβ-ΔC-FLAG (middle), and Alcγ-ΔC-FLAG (right) in the presence of wild-type PS1 (upper) or PS1 D385A mutant (lower) were subjected to *in vitro* **γ**-secretase assay to generate Alc ICD-ΔC-FLAG, which was recovered by immunoprecipitation with anti-FLAG antibody and analyzed with MALDI-TOF/MS. The major product cleaved at the ε1 site (closed arrowhead) and minor products cleaved at ε2 and ε3 sites (open arrowheads) are indicated.
(TIF)

Figure S3 **Identification of major and minor ε-cleavage sites of Alcadeins.** Amino acid sequences of Alcα ICD-ΔC-FLAG generated from Alcα-ΔC-FLAG (**A**), Alcβ ICD-ΔC-FLAG generated from Alcβ-ΔC-FLAG (**B**), and Alcγ ICD-ΔC-FLAG generated from Alcγ-ΔC-FLAG (**C**) were determined by MALDI-MS/MS analysis. Left panels show the major Alc ICD-ΔC-FLAG product with N-terminal ε1 site, right (A) and middle panels (B and C) show minor products with N-terminal ε2 sites, and right panels (B and C) show additional minor products with N-terminal ε3 sites. Representative MS spectra of Alc ICD-ΔC-FLAG are shown in Fig. 1A, and the amino acid sequences determined by this study are indicated in Fig. 1B. Amino acid sequence “DYKDDDDK” indicates FLAG sequence.
(TIF)

Figure S4 **Comparison of p3-Alc species generated by membrane incubation (** ***in vitro*** **γ-secretase assay) with those secreted by cells.** Comparison of representative MS spectra of p3-Alc species secreted into culture medium by cells expressing Alc CTF (Medium) with those generated by *in vitro* γ-secretase assay with membrane fractions prepared from cells expressing Alc CTF (Membrane incubation). The peak area of minor p3-Alc (γ2, open arrowheads) was compared with that of major p3-Alc (γ1, closed arrowheads), and the minor/major (γ2/γ1) ratios are indicated (right). To examine the background signals, MS spectra of *in vitro* γ-secretase assay without incubation are shown (Membrane incubation (-)). **A.** Spectra of p3-Alcα (left panels), and the minor/major ratio (p3-Alcα2N+38/p3-Alcα2N+35) in medium and the ratio (p3-Alcα38/p3-Alcα35) generated by *in vitro* γ-secretase assay (right graph) are shown. In the *in vitro* γ-secretase assay, p3-Alcα species were predominantly generated, while p3-Alcα2N+ species were predominantly secreted into the culture medium by cells. Thus, we compared the γ2/γ1 ratios between the p3-Alcα2N+38/p3-Alcα2N+35 ratio in media and the p3-Alcα38/p3-Alcα35 ratio in membrane incubation. **B.** Spectra of p3-Alcβ (left panels), and the minor/major ratios (p3-Alcβ37/p3-Alcβ40) secreted into medium and generated by *in vitro* γ-secretase assay (right graph) are shown. **C.** Spectra of p3-Alcγ (left panels), and the minor/major ratios (p3-Alcγ34/p3-Alcγ31) secreted into medium and generated by *in vitro* γ-secretase assay (right graph) are shown. (**A–C**) Statistical analysis was performed using Student’s t test (mean ± S.E., n = 4). No significant difference between medium and *in vitro* γ-secretase assay was observed.
(TIF)

Figure S5 **Displacement of the intramembrane γ- and ε-sites of Alcα, Alcβ Alcγ and APP in cells expressing FAD-linked mutations of PS1.** (**A–C**) Representative MS spectra of p3-Alc (upper) secreted by cells expressing wild-type PS1 and FAD-linked mutants of PS1, and Alc ICD (lower) generated by *in vitro* γ-secretase assay with membranes from the same cells. **A.** The p3-Alcα species secreted by HEK293 cells expressing Alcα-ΔC-FLAG were immunoprecipitated and subjected to MALDI-TOF/MS analysis. The Alcα ICD-ΔC-FLAG species generated by *in vitro* γ-secretase assay were immunoprecipitated and analyzed by MALD-TOF/MS analysis. Spectra of the minor product p3-Alcα38 (γ2) are enlarged in windows, in which intensity of 300 on the y-axis corresponds to 0.03 in the original panels. **B.** The p3-Alcβ species secreted by HEK293 cells expressing Alcβ-ΔC-FLAG were immunoprecipitated and subjected to MALDI-TOF/MS analysis. The Alcβ ICD-ΔC-FLAG species generated by *in vitro* γ-secretase assay were immunoprecipitated and analyzed by MALD-TOF/MS analysis. Spectra of minor sites (ε2 and ε3) are enlarged in windows in which intensities of 1200 and 800 on the y-axis correspond to 0.12 and 0.08, respectively, in the original panels. **C.** The p3-Alcγ species secreted by HEK293 cells expressing Alcγ-ΔC-FLAG were immunoprecipitated and subjected to MALDI-TOF/MS analysis. The Alcγ ICD-ΔC-FLAG species generated by *in vitro* γ-secretase assay were immunoprecipitated and analyzed by MALD-TOF/MS analysis. (**A–C**) Closed arrowheads indicate major γ- or ε-site cleaved products: p3-Alcα2N+35 (panel A upper) and Alcα ICDε1 (panel A lower), p3-Alcβ40 (panel B upper) and Alcβ ICD-ε1 (panel B lower), and p3-Alcγ31 (panel C upper) and Alcγ ICD-ε1 (panel C lower). Open arrowheads indicate minor γ- or ε-cleaved products: p3-Alcα2N+38 (panel A upper) and Alcα ICD-ε2 (panel A lower); p3-Alcβ37 (panel B upper), Alcβ ICD-ε2, and Alcβ ICD-ε3 (panel B lower); and p3-Alcγ34 (panel C upper), Alcγ ICD-ε2, and Alcγ ICD-ε3 (panel C lower). **D.** Representative MS spectra of AICD. The AICD-FLAG generated by *in vitro* γ-secretase assay with membranes from HEK293 cells expressing CTFβ/C99-FLAG were immunoprecipitated with anti-FLAG antibody and analyzed by MALD-TOF/MS analysis. Closed arrowheads indicate major ε1-cleaved products, and open arrowheads indicate minor ε2-cleaved products.
(TIF)

Figure S6 **Correlation between minor/major ratios of γ-cleavage products and ε-cleavage products.** Covariant analysis of γ2/γ1 ratio with the ratios of certain minor ε2 orε3 products to major ε1 products was performed. Graphs showing the relationships between the ratio of p3-Alcα γ2/γ1 to Alcα ICD ε2/ε1 ratio (**A**), the ratio of p3-Alcβ γ2/γ1 to Alcβ ICD ε2/ε1 and ε3/ε1 ratios (**B**), the ratio of p3-Alcγ γ2/γ1 to Alcγ ICD ε2/ε1 and ε3/ε1 ratios (**C**), and the ratio Aβ42/Aβ40 to AICD ε2/ε1 ratio (**D**). wt, wild-type PS1; A434C, L166P, and R278T are FAD-linked PS1 mutants (see Figs. 3–5). R^2^, correlation coefficient.
(TIF)

Figure S7 **Schematic structure of Alc-ΔC proteins with physiological ε-cleavage sites.** A. The cytoplasmic region of Alcα was truncated at the indicated major ε1 and minor ε2 sites and fused to a signal peptide (SP) sequence at the N terminal through a Met+Ala sequence composed of “2N+” species. Amino acid numbering corresponds to human Alcadeinα1 (971 amino acids). The primary α-cleavage site is indicated with a gray arrowhead, and the cleavage indicated with a broken-line arrowhead generates Alcα CTF. Positions of γ-cleavage sites are indicated with open arrowheads, and ε-sites are indicated with closed arrowheads (the larger arrowhead indicates the major ε-site, and the smaller indicates the minor ε-site). **B.** The cytoplasmic region of Alcβ was truncated at the indicated major ε1 and minor ε2 and ε3 sites and fused to a signal peptide (SP) sequence at the N terminal. Amino acid numbering corresponds to human Alcadein β (956 amino acids). The primary α-cleavage site is indicated with a gray arrowhead, and the cleavage indicated with a broken-line arrowhead generates Alcβ CTF. Positions of γ-cleavage sites are indicated with open arrowheads, and ε-sites are indicated with closed arrowheads (the larger arrowhead indicates the major ε-site, and the smaller two indicate minor ε-sites). **C.** The cytoplasmic region of Alcγ was truncated at the indicated major ε1 and minor ε2 and ε3 sites and fused to a signal peptide (SP) sequence at the N terminal. Amino acid numbering corresponds to human Alcadein γ (955 amino acids). The primary α-cleavage site is indicated with a gray arrowhead, and the cleavage indicated with a broken-line arrowhead generates Alc γ CTF. Positions of γ-cleavage sites are indicated with open arrowheads, and ε-sites are indicated with closed arrowheads (the larger arrowhead indicates the major ε-site, and the smaller two indicate minor ε-sites).
(TIF)

Figure S8 **Identification of p3-Alcβ species secreted by cells.** In Fig. 6B, the immunoprecipitation-TOF-MS study using the media of cells expressing Alcβ CTF with C-terminal truncated ε-site presented complex spectra. To identify p3-Alcβ species secreted by cells, mock media derived from cells without expression of Alcβ CTF was also analyzed, and the spectra were compared to those of cells expressing Alcβ CTF. The major p3-Alcβ40 (γ1) and minor p3-Alcβ37 (γ2) products are indicated with arrowheads. Other MS signals are not products derived from Alcβ CTF.
(TIF)

Figure S9 **Quantitative accuracy of immunoprecipitation-mass spectrometric analysis in the presence of another peptide (I).** Endogenously generated p3-Alcα 2N+35 in the presence of increased amount of synthetic p3-Alcα35 peptide was subjected to immunoprecipitation with UT135 and analyzed with MALDI-TOF/MS. **A.** Amino acid sequence of p3-Alcα2N+35 and p3-Alcα35. **B.** Representative immunoprecipitation-mass spectra of endogenous and synthetic p3-Alcα peptides. To fixed volume (2 mL) of cultured medium of HEK293 cells expressing Alcα, indicated amount (0, 0.5, 1.0, 2.0 and 4.0 ng) of synthetic p3-Alcα35 peptide was added, and subjected to immunoprecipitation. The cells secrete p3-Alcα 2N+35 (closed arrowhead) largely with small amount of p3-Alcα35 (open arrowhead) (left panel, 0 ng of synthetic peptide). **C.** Quantitative accuracy of the ratio of p3-Alcα35/p3-Alcα 2N+35. The relationship of area ratios of p3-Alcα35/p3-Alcα 2N+35 in the presence of various amounts of synthetic p3-Alcα35 peptide were analyzed. The endogenous p3-Alcα 2N+35 levels are not affected in the presence of increased amount of synthetic p3-Alcα35 peptide (B), and the p3-Alcα35/p3-Alcα 2N+35 ratio increased proportionally with the increased amount of synthetic p3-Alcα35 peptide (R^2^ = 0.99938 in C), indicating the quantification of a specific peptide is not affected in the presence of increased amounts of another peptide in this immunoprecipitation-mass spectrometric analysis.
(TIF)

Figure S10 **Quantitative accuracy of immunoprecipitation-mass spectrometric analysis in the presence of another peptide (II).** Synthetic p3-Alcβ37 and p3-Alcβ40 were subjected to immunoprecipitation with UT143 and analyzed with MALDI-TOF/MS. **A.** Amino acid sequence of p3-Alcβ37 and p3-Alcβ40. **B–C.** Representative immunoprecipitation-mass spectra of synthetic p3-Alcβ peptides in PBS (2 mL) containing 0.1% (W/V) bovine serum albumin (B) or cultured medium (2 mL) of mock HEK293 cells (C). To the fixed amount (1 ng) of synthetic p3-Alcβ37 peptide (open arrowhead) indicated amount (0, 0.5, 1.0, 2.0 and 4.0 ng) of synthetic p3-Alcβ40 peptide (closed arrowhead) was added, and subjected to immunoprecipitation. The cells don't secrete p3-Alcβ species but show non-specific products as signals, which are detectable in C, but not in B, along with synthetic p3-Alcβ37 (open arrowhead) and p3-Alcβ40 (closed arrowhead). **D–E.** Quantitative accuracy of the ratio of p3-Alcβ40/p3-Alcβ37. The relationship of area ratios of p3-Alcβ40/p3-Alcβ37 with various amounts of synthetic p3-Alcβ40 peptide were analyzed (panel D indicates the result of B, and panel E indicates the result of C). The synthetic p3-Alcβ37 levels are not affected in the presence of increased amount of synthetic p3-Alcβ40 peptide (B) and unknown immunoprecipitates (C), and the p3-Alcβ40/p3-Alcβ37 ratio increased proportionally with the increased amount of synthetic p3-Alcβ40 peptide (R^2^ = 0.99866 in D and R^2^ = 0.99861 in E), indicating the quantification of a specific peptide is not affected in the presence of increased amounts of another peptide in this immunoprecipitation-mass spectrometric analysis.
(TIF)

Table S1 **Molecular masses observed by TOF/MS analysis and expected.** Molecular masses (Da) of Alc ICD-ΔC-FLAG peptides (**upper**) and p3-Alcs (**lower**) generated by *in vitro* γ-secretase assay with cell membranes. The p3-Alcα peptide products γ1/35 and γ2/38 indicate p3-Alcα35 and p3-Alcα38, respectively, but not p3-Alcα2N+35 and p3-Alcα2N+38, which are secreted by cultured cells [17], because the *in vitro* γ-secretase assay with cultured cell membranes generates dominantly p3-Alcα species but not p3-Alcα2N+ species (see **Fig. S4A**).
(TIF)

Table S2 **Net Aβ values quantified with sELISA.** Medium Aβ40 and Aβ42 values of the studies indicated in Fig. 2D were quantified with sELISA [27], and the average values are summarized with standard deviation (n = 4).
(TIF)
