# Supplementary figures and images for: Mechanism of Intramembrane Cleavage of Alcadeins by γ-Secretase
Source: PLoS One. 2013 Apr 26;8(4):e62431. doi: 10.1371/journal.pone.0062431 (PMC3637299; doi:10.1371/journal.pone.0062431)

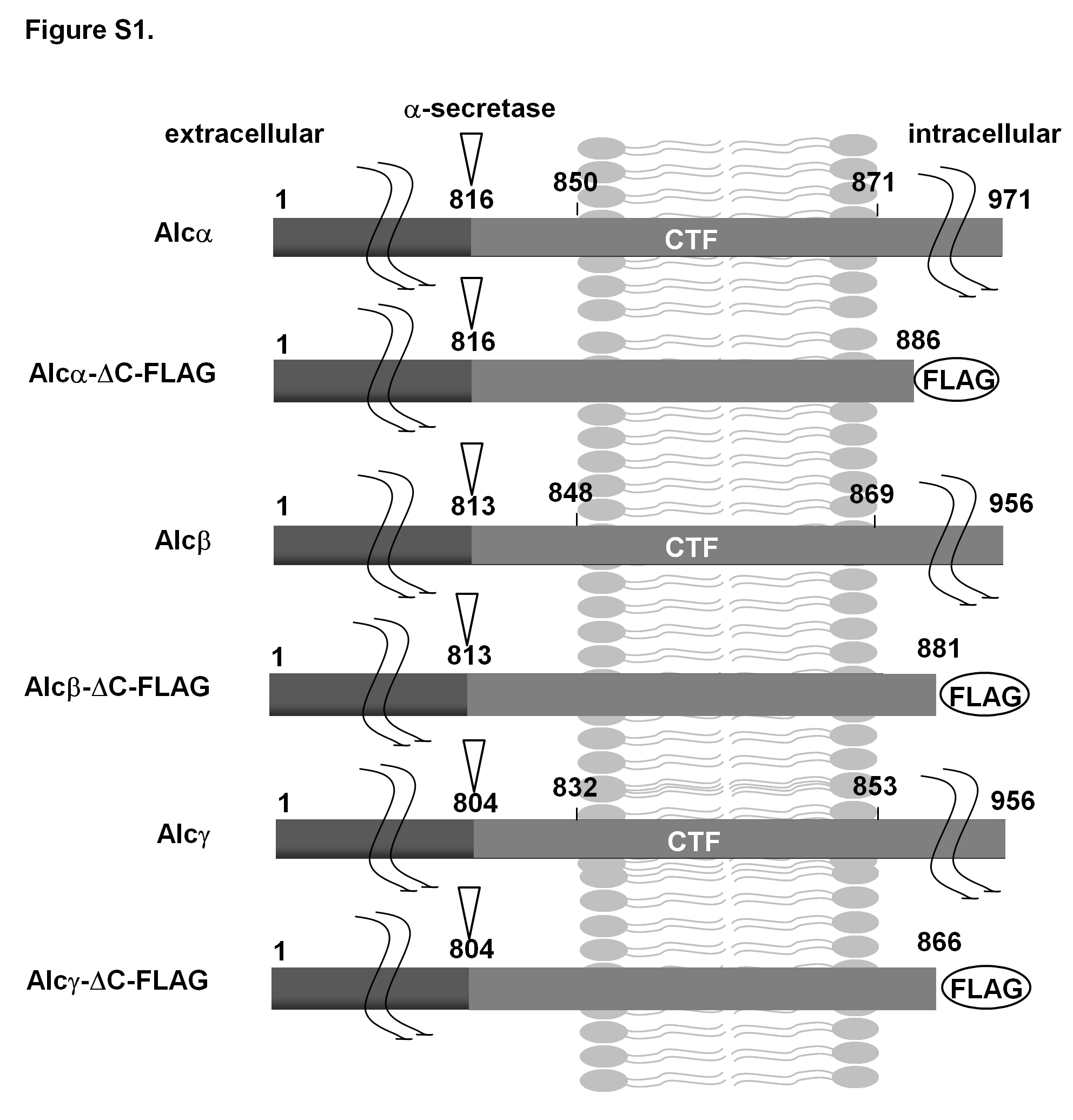

Supplement: Figure S1 — Schematic structure of Alc-ΔC-FLAG fusion proteins used for the in vitro γ-secretase assay to determine ε-cleavage sites. Cytoplasmic regions of Alcα, Alcβ and Alcγ were truncated at the indicated positions and fused to FLAG-tag sequence. Amino acid numbering corresponds to human Alcadein α1 (971 amino acids), Alcadein β (956 mino acids), and Alcadein γ (956 amino acids) [16]. Primary α-cleavage sites are indicated with open arrowheads. CTF (light gray shading on Alc proteins), C-terminal region of Alc cleaved by α-secretase. (TIF) [file pone.0062431.s001.tif]

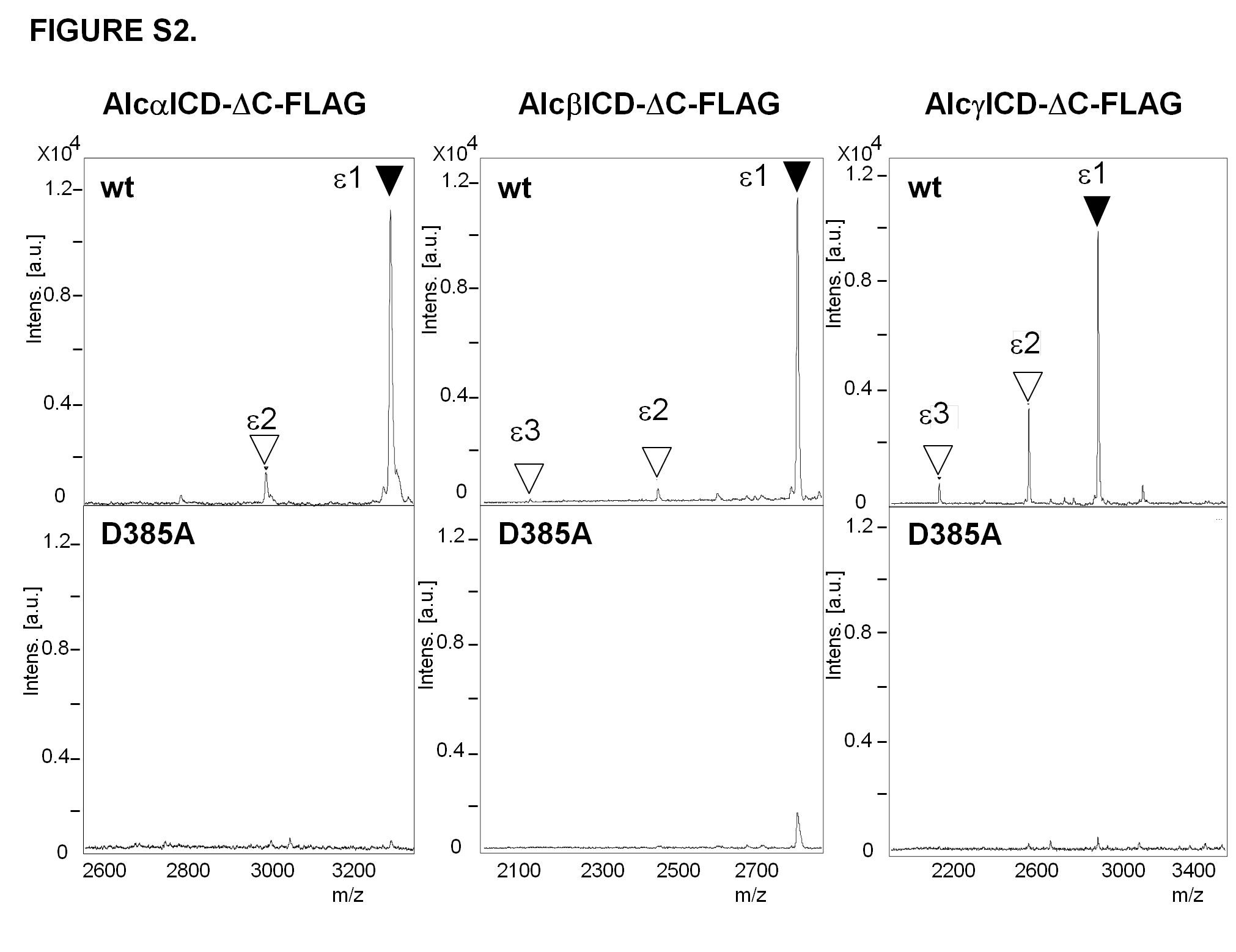

Supplement: Figure S2 — Representative MS spectra of Alc ICD-ΔC-FLAG generated by in vitro γ-secretase assay with membranes derived from cells expressing wild-type PS1 or the dominant-negative PS1 mutant D385A. Membranes from cells expressing Alcα-ΔC-FLAG (left), Alcβ-ΔC-FLAG (middle), and Alcγ-ΔC-FLAG (right) in the presence of wild-type PS1 (upper) or PS1 D385A mutant (lower) were subjected to in vitro γ-secretase assay to generate Alc ICD-ΔC-FLAG, which was recovered by immunoprecipitation with anti-FLAG antibody and analyzed with MALDI-TOF/MS. The major product cleaved at the ε1 site (closed arrowhead) and minor products cleaved at ε2 and ε3 sites (open arrowheads) are indicated. (TIF) [file pone.0062431.s002.tif]

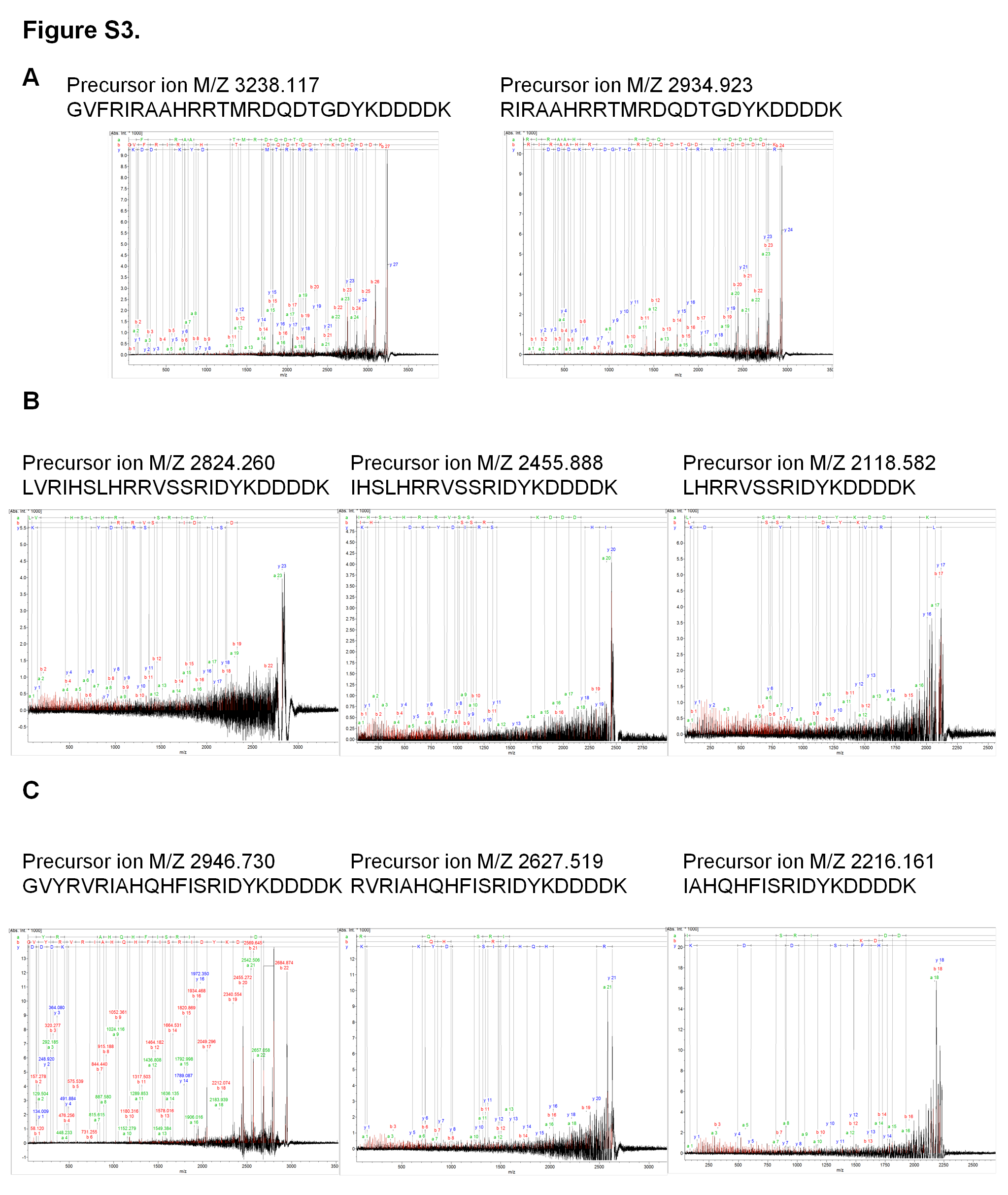

Supplement: Figure S3 — Identification of major and minor ε-cleavage sites of Alcadeins. Amino acid sequences of Alcα ICD-ΔC-FLAG generated from Alcα-ΔC-FLAG (A), Alcβ ICD-ΔC-FLAG generated from Alcβ-ΔC-FLAG (B), and Alcγ ICD-ΔC-FLAG generated from Alcγ-ΔC-FLAG (C) were determined by MALDI-MS/MS analysis. Left panels show the major Alc ICD-ΔC-FLAG product with N-terminal ε1 site, right (A) and middle panels (B and C) show minor products with N-terminal ε2 sites, and right panels (B and C) show additional minor products with N-terminal ε3 sites. Representative MS spectra of Alc ICD-ΔC-FLAG are shown in Fig. 1A, and the amino acid sequences determined by this study are indicated in Fig. 1B. Amino acid sequence “DYKDDDDK” indicates FLAG sequence. (TIF) [file pone.0062431.s003.tif]

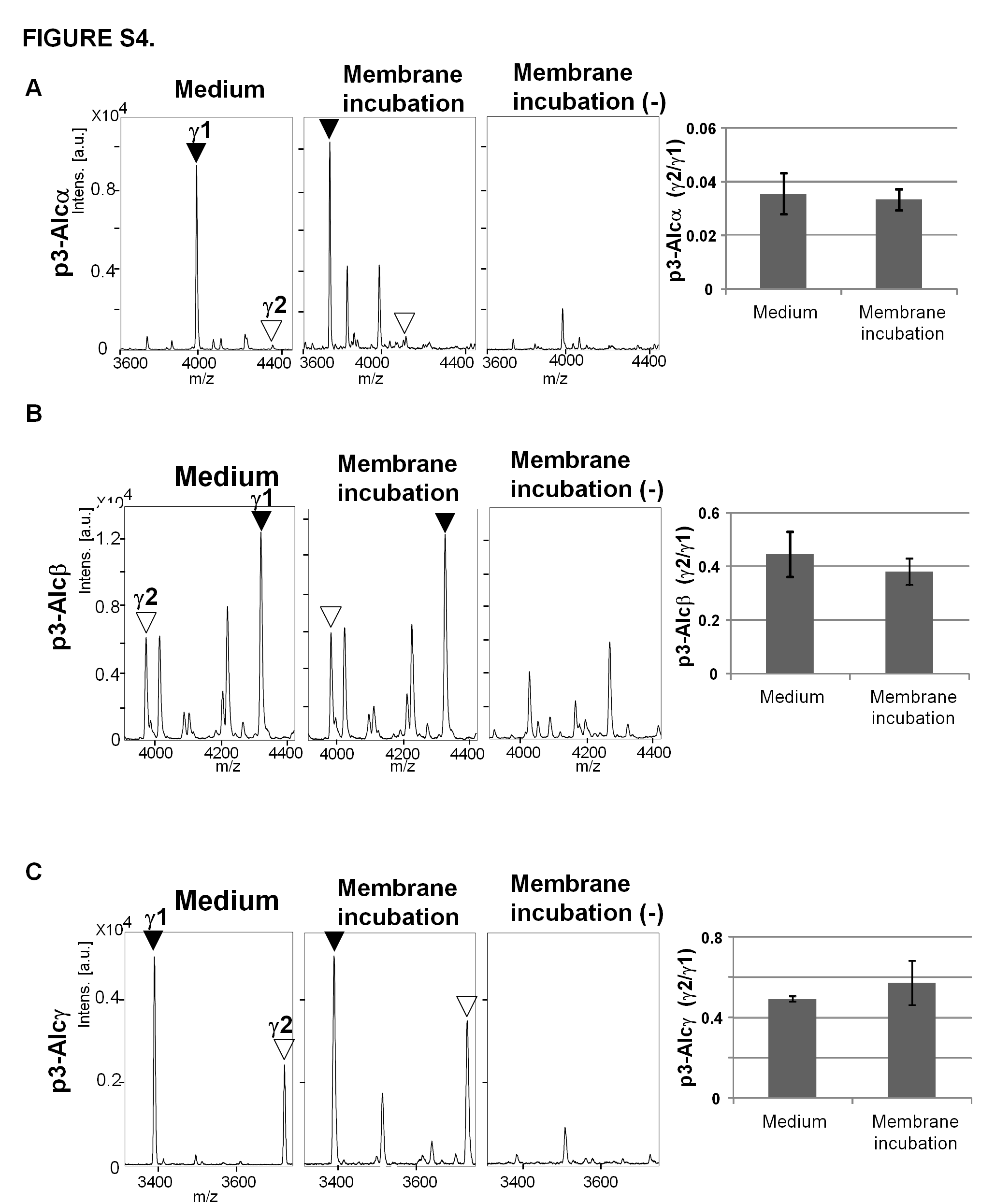

Supplement: Figure S4 — Comparison of p3-Alc species generated by membrane incubation ( in vitro γ-secretase assay) with those secreted by cells. Comparison of representative MS spectra of p3-Alc species secreted into culture medium by cells expressing Alc CTF (Medium) with those generated by in vitro γ-secretase assay with membrane fractions prepared from cells expressing Alc CTF (Membrane incubation). The peak area of minor p3-Alc (γ2, open arrowheads) was compared with that of major p3-Alc (γ1, closed arrowheads), and the minor/major (γ2/γ1) ratios are indicated (right). To examine the background signals, MS spectra of in vitro γ-secretase assay without incubation are shown (Membrane incubation (-)). A. Spectra of p3-Alcα (left panels), and the minor/major ratio (p3-Alcα2N+38/p3-Alcα2N+35) in medium and the ratio (p3-Alcα38/p3-Alcα35) generated by in vitro γ-secretase assay (right graph) are shown. In the in vitro γ-secretase assay, p3-Alcα species were predominantly generated, while p3-Alcα2N+ species were predominantly secreted into the culture medium by cells. Thus, we compared the γ2/γ1 ratios between the p3-Alcα2N+38/p3-Alcα2N+35 ratio in media and the p3-Alcα38/p3-Alcα35 ratio in membrane incubation. B. Spectra of p3-Alcβ (left panels), and the minor/major ratios (p3-Alcβ37/p3-Alcβ40) secreted into medium and generated by in vitro γ-secretase assay (right graph) are shown. C. Spectra of p3-Alcγ (left panels), and the minor/major ratios (p3-Alcγ34/p3-Alcγ31) secreted into medium and generated by in vitro γ-secretase assay (right graph) are shown. (A–C) Statistical analysis was performed using Student’s t test (mean ± S.E., n = 4). No significant difference between medium and in vitro γ-secretase assay was observed. (TIF) [file pone.0062431.s004.tif]

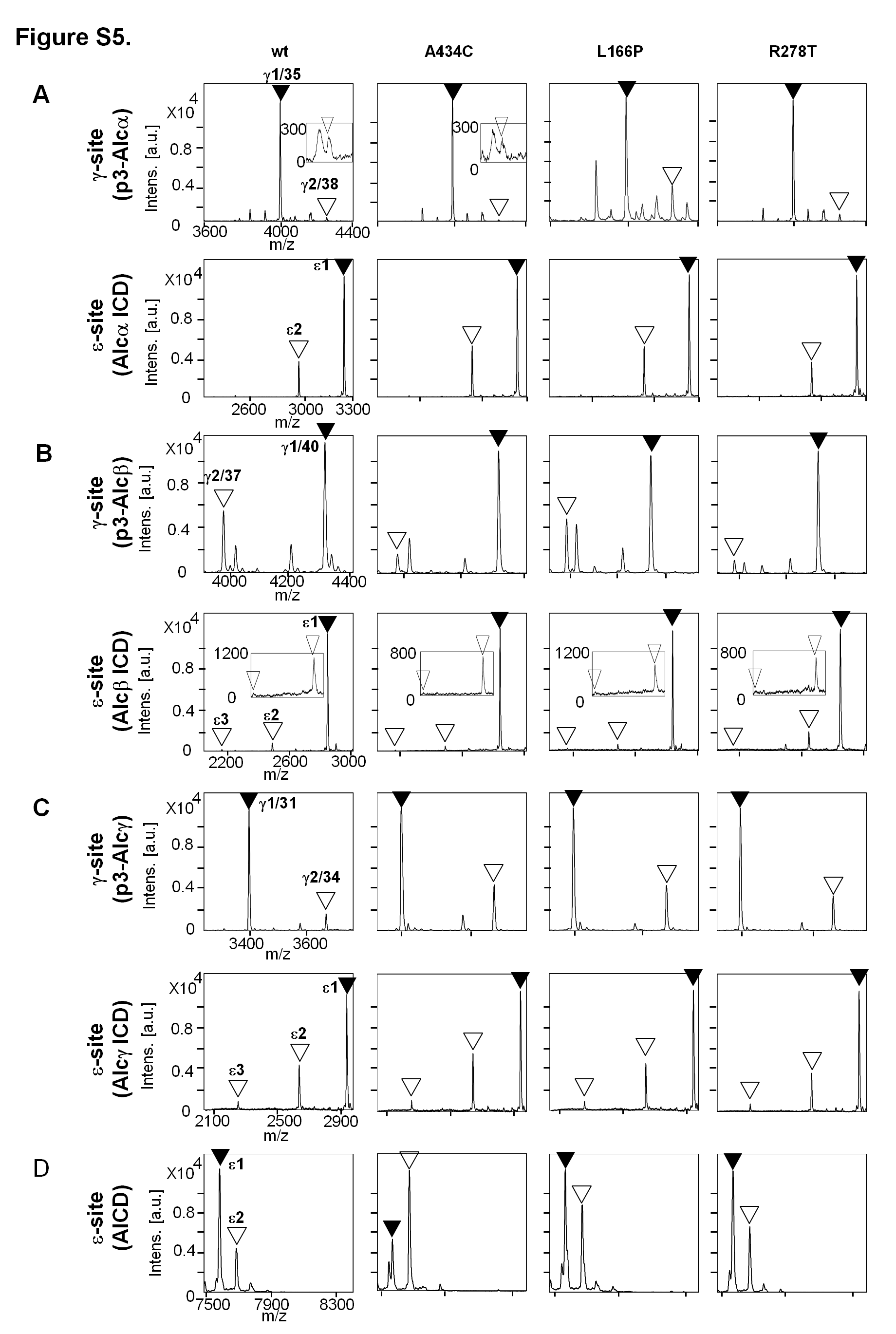

Supplement: Figure S5 — Displacement of the intramembrane γ- and ε-sites of Alcα, Alcβ Alcγ and APP in cells expressing FAD-linked mutations of PS1. (A–C) Representative MS spectra of p3-Alc (upper) secreted by cells expressing wild-type PS1 and FAD-linked mutants of PS1, and Alc ICD (lower) generated by in vitro γ-secretase assay with membranes from the same cells. A. The p3-Alcα species secreted by HEK293 cells expressing Alcα-ΔC-FLAG were immunoprecipitated and subjected to MALDI-TOF/MS analysis. The Alcα ICD-ΔC-FLAG species generated by in vitro γ-secretase assay were immunoprecipitated and analyzed by MALD-TOF/MS analysis. Spectra of the minor product p3-Alcα38 (γ2) are enlarged in windows, in which intensity of 300 on the y-axis corresponds to 0.03 in the original panels. B. The p3-Alcβ species secreted by HEK293 cells expressing Alcβ-ΔC-FLAG were immunoprecipitated and subjected to MALDI-TOF/MS analysis. The Alcβ ICD-ΔC-FLAG species generated by in vitro γ-secretase assay were immunoprecipitated and analyzed by MALD-TOF/MS analysis. Spectra of minor sites (ε2 and ε3) are enlarged in windows in which intensities of 1200 and 800 on the y-axis correspond to 0.12 and 0.08, respectively, in the original panels. C. The p3-Alcγ species secreted by HEK293 cells expressing Alcγ-ΔC-FLAG were immunoprecipitated and subjected to MALDI-TOF/MS analysis. The Alcγ ICD-ΔC-FLAG species generated by in vitro γ-secretase assay were immunoprecipitated and analyzed by MALD-TOF/MS analysis. (A–C) Closed arrowheads indicate major γ- or ε-site cleaved products: p3-Alcα2N+35 (panel A upper) and Alcα ICDε1 (panel A lower), p3-Alcβ40 (panel B upper) and Alcβ ICD-ε1 (panel B lower), and p3-Alcγ31 (panel C upper) and Alcγ ICD-ε1 (panel C lower). Open arrowheads indicate minor γ- or ε-cleaved products: p3-Alcα2N+38 (panel A upper) and Alcα ICD-ε2 (panel A lower); p3-Alcβ37 (panel B upper), Alcβ ICD-ε2, and Alcβ ICD-ε3 (panel B lower); and p3-Alcγ34 (panel C upper), Alcγ ICD-ε2, and Alcγ ICD-ε3 (panel C lower). D. [file pone.0062431.s005.tif]

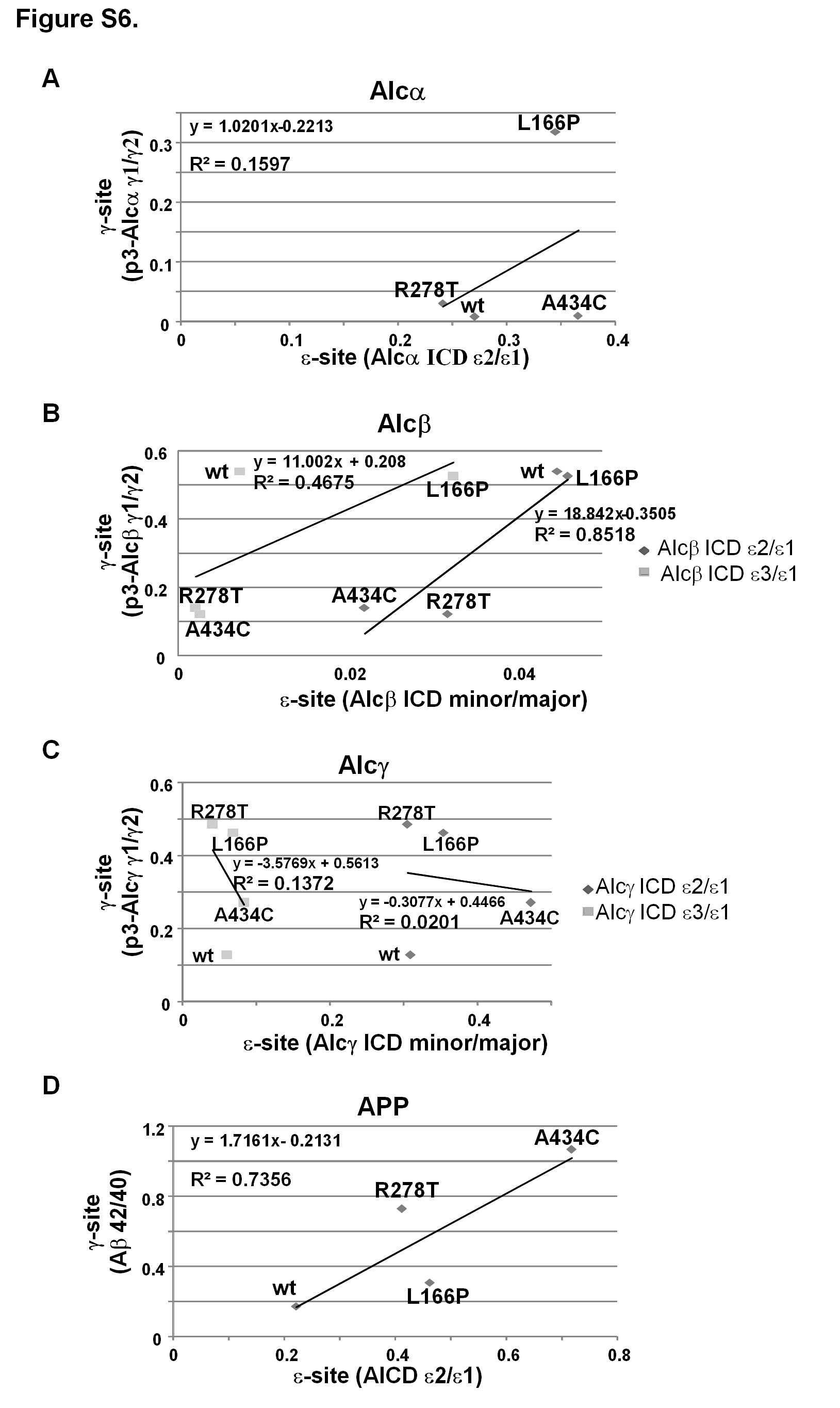

Supplement: Figure S6 — Correlation between minor/major ratios of γ-cleavage products and ε-cleavage products. Covariant analysis of γ2/γ1 ratio with the ratios of certain minor ε2 orε3 products to major ε1 products was performed. Graphs showing the relationships between the ratio of p3-Alcα γ2/γ1 to Alcα ICD ε2/ε1 ratio (A), the ratio of p3-Alcβ γ2/γ1 to Alcβ ICD ε2/ε1 and ε3/ε1 ratios (B), the ratio of p3-Alcγ γ2/γ1 to Alcγ ICD ε2/ε1 and ε3/ε1 ratios (C), and the ratio Aβ42/Aβ40 to AICD ε2/ε1 ratio (D). wt, wild-type PS1; A434C, L166P, and R278T are FAD-linked PS1 mutants (see Figs. 3–5). R2, correlation coefficient. (TIF) [file pone.0062431.s006.tif]

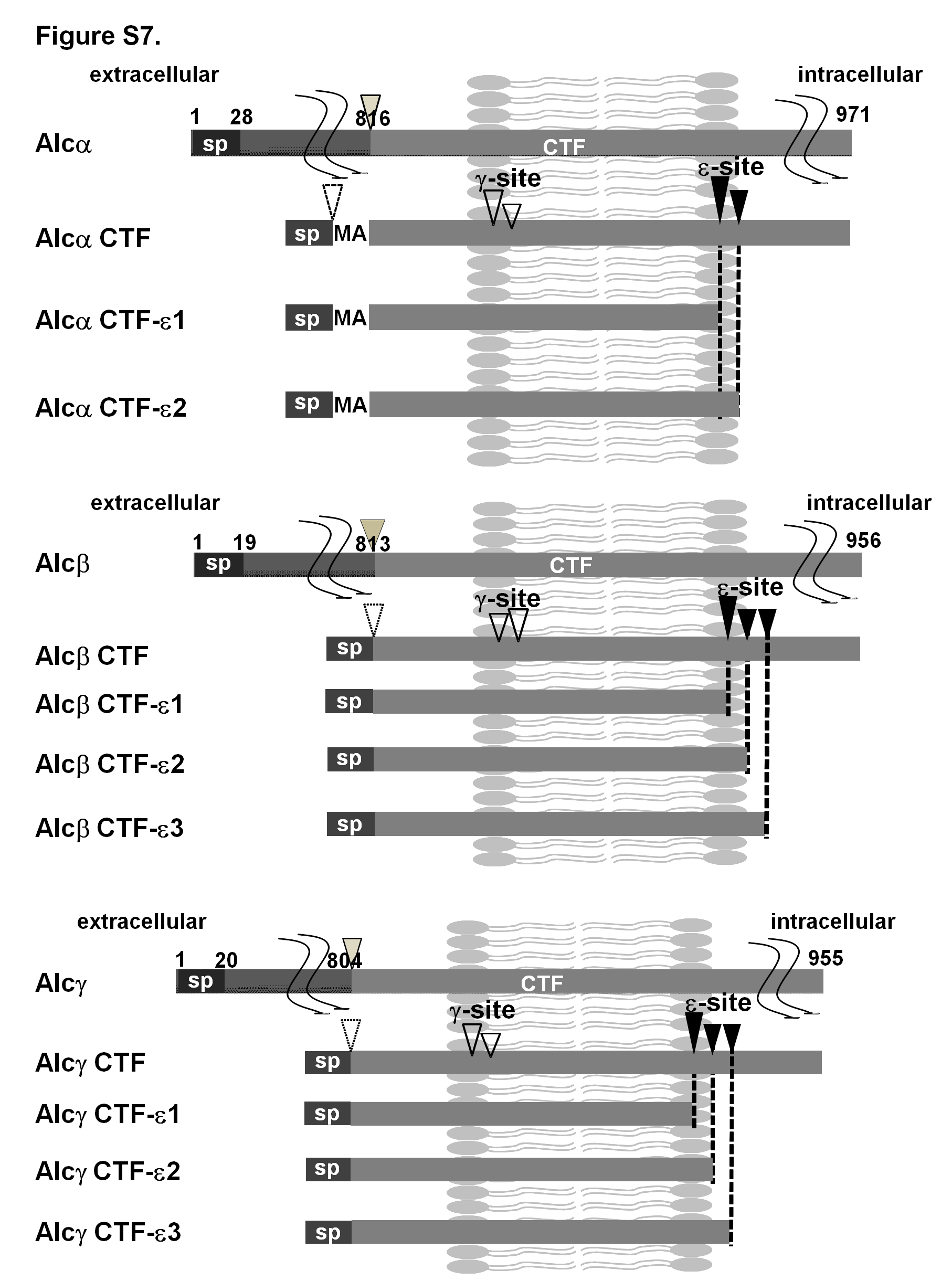

Supplement: Figure S7 — Schematic structure of Alc-ΔC proteins with physiological ε-cleavage sites. A. The cytoplasmic region of Alcα was truncated at the indicated major ε1 and minor ε2 sites and fused to a signal peptide (SP) sequence at the N terminal through a Met+Ala sequence composed of “2N+” species. Amino acid numbering corresponds to human Alcadeinα1 (971 amino acids). The primary α-cleavage site is indicated with a gray arrowhead, and the cleavage indicated with a broken-line arrowhead generates Alcα CTF. Positions of γ-cleavage sites are indicated with open arrowheads, and ε-sites are indicated with closed arrowheads (the larger arrowhead indicates the major ε-site, and the smaller indicates the minor ε-site). B. The cytoplasmic region of Alcβ was truncated at the indicated major ε1 and minor ε2 and ε3 sites and fused to a signal peptide (SP) sequence at the N terminal. Amino acid numbering corresponds to human Alcadein β (956 amino acids). The primary α-cleavage site is indicated with a gray arrowhead, and the cleavage indicated with a broken-line arrowhead generates Alcβ CTF. Positions of γ-cleavage sites are indicated with open arrowheads, and ε-sites are indicated with closed arrowheads (the larger arrowhead indicates the major ε-site, and the smaller two indicate minor ε-sites). C. The cytoplasmic region of Alcγ was truncated at the indicated major ε1 and minor ε2 and ε3 sites and fused to a signal peptide (SP) sequence at the N terminal. Amino acid numbering corresponds to human Alcadein γ (955 amino acids). The primary α-cleavage site is indicated with a gray arrowhead, and the cleavage indicated with a broken-line arrowhead generates Alc γ CTF. Positions of γ-cleavage sites are indicated with open arrowheads, and ε-sites are indicated with closed arrowheads (the larger arrowhead indicates the major ε-site, and the smaller two indicate minor ε-sites). (TIF) [file pone.0062431.s007.tif]

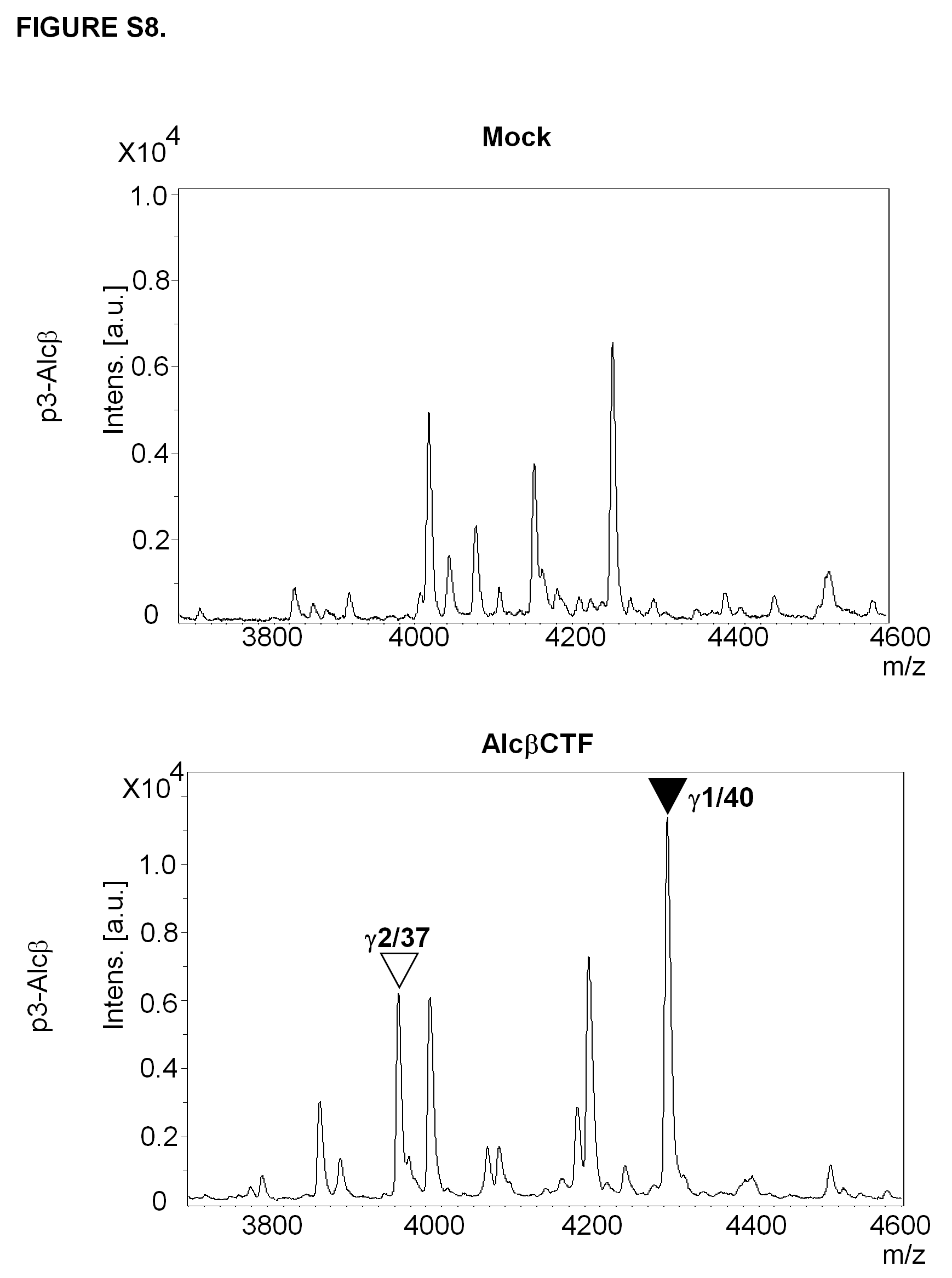

Supplement: Figure S8 — Identification of p3-Alcβ species secreted by cells. In Fig. 6B, the immunoprecipitation-TOF-MS study using the media of cells expressing Alcβ CTF with C-terminal truncated ε-site presented complex spectra. To identify p3-Alcβ species secreted by cells, mock media derived from cells without expression of Alcβ CTF was also analyzed, and the spectra were compared to those of cells expressing Alcβ CTF. The major p3-Alcβ40 (γ1) and minor p3-Alcβ37 (γ2) products are indicated with arrowheads. Other MS signals are not products derived from Alcβ CTF. (TIF) [file pone.0062431.s008.tif]

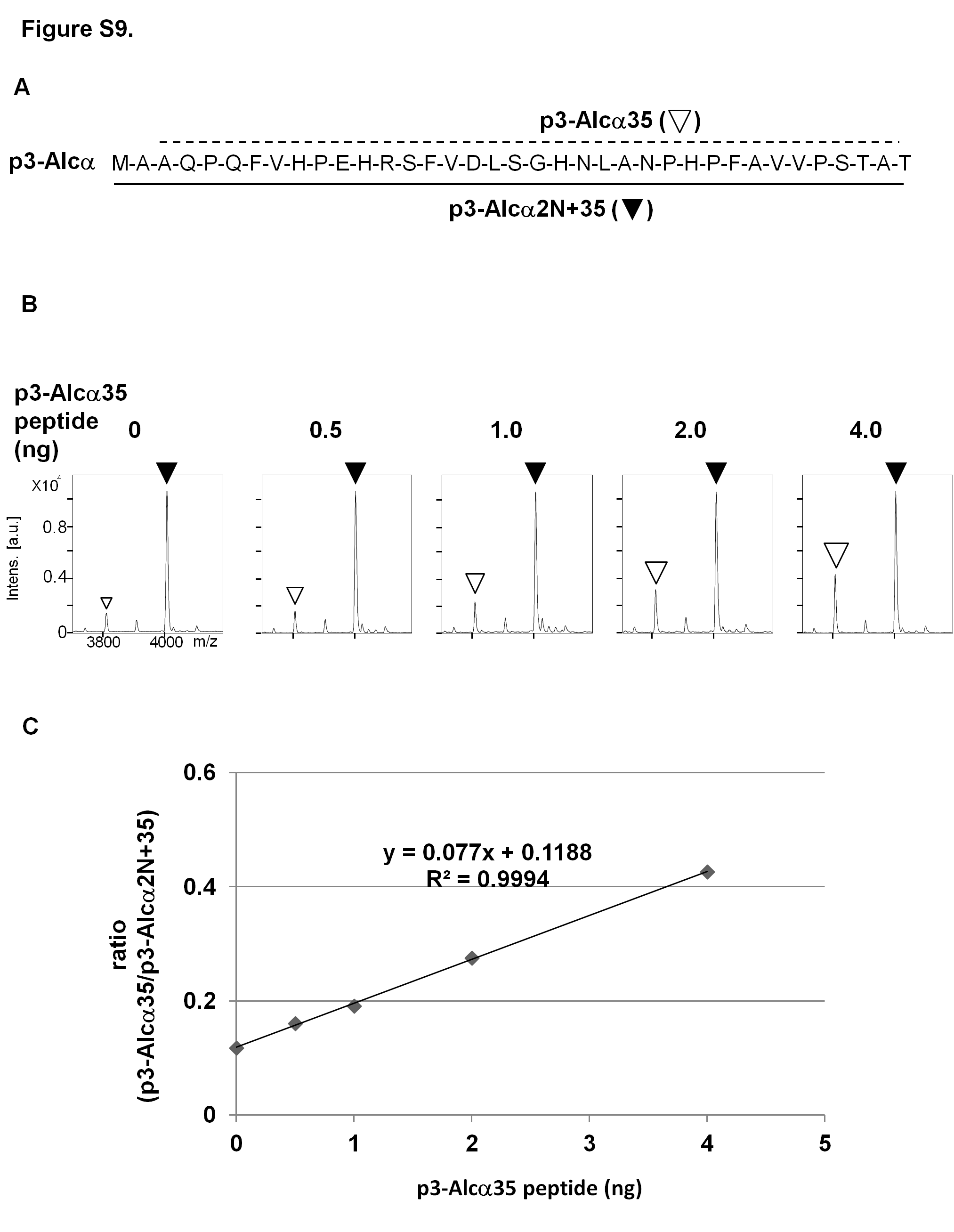

Supplement: Figure S9 — Quantitative accuracy of immunoprecipitation-mass spectrometric analysis in the presence of another peptide (I). Endogenously generated p3-Alcα 2N+35 in the presence of increased amount of synthetic p3-Alcα35 peptide was subjected to immunoprecipitation with UT135 and analyzed with MALDI-TOF/MS. A. Amino acid sequence of p3-Alcα2N+35 and p3-Alcα35. B. Representative immunoprecipitation-mass spectra of endogenous and synthetic p3-Alcα peptides. To fixed volume (2 mL) of cultured medium of HEK293 cells expressing Alcα, indicated amount (0, 0.5, 1.0, 2.0 and 4.0 ng) of synthetic p3-Alcα35 peptide was added, and subjected to immunoprecipitation. The cells secrete p3-Alcα 2N+35 (closed arrowhead) largely with small amount of p3-Alcα35 (open arrowhead) (left panel, 0 ng of synthetic peptide). C. Quantitative accuracy of the ratio of p3-Alcα35/p3-Alcα 2N+35. The relationship of area ratios of p3-Alcα35/p3-Alcα 2N+35 in the presence of various amounts of synthetic p3-Alcα35 peptide were analyzed. The endogenous p3-Alcα 2N+35 levels are not affected in the presence of increased amount of synthetic p3-Alcα35 peptide (B), and the p3-Alcα35/p3-Alcα 2N+35 ratio increased proportionally with the increased amount of synthetic p3-Alcα35 peptide (R2 = 0.99938 in C), indicating the quantification of a specific peptide is not affected in the presence of increased amounts of another peptide in this immunoprecipitation-mass spectrometric analysis. (TIF) [file pone.0062431.s009.tif]

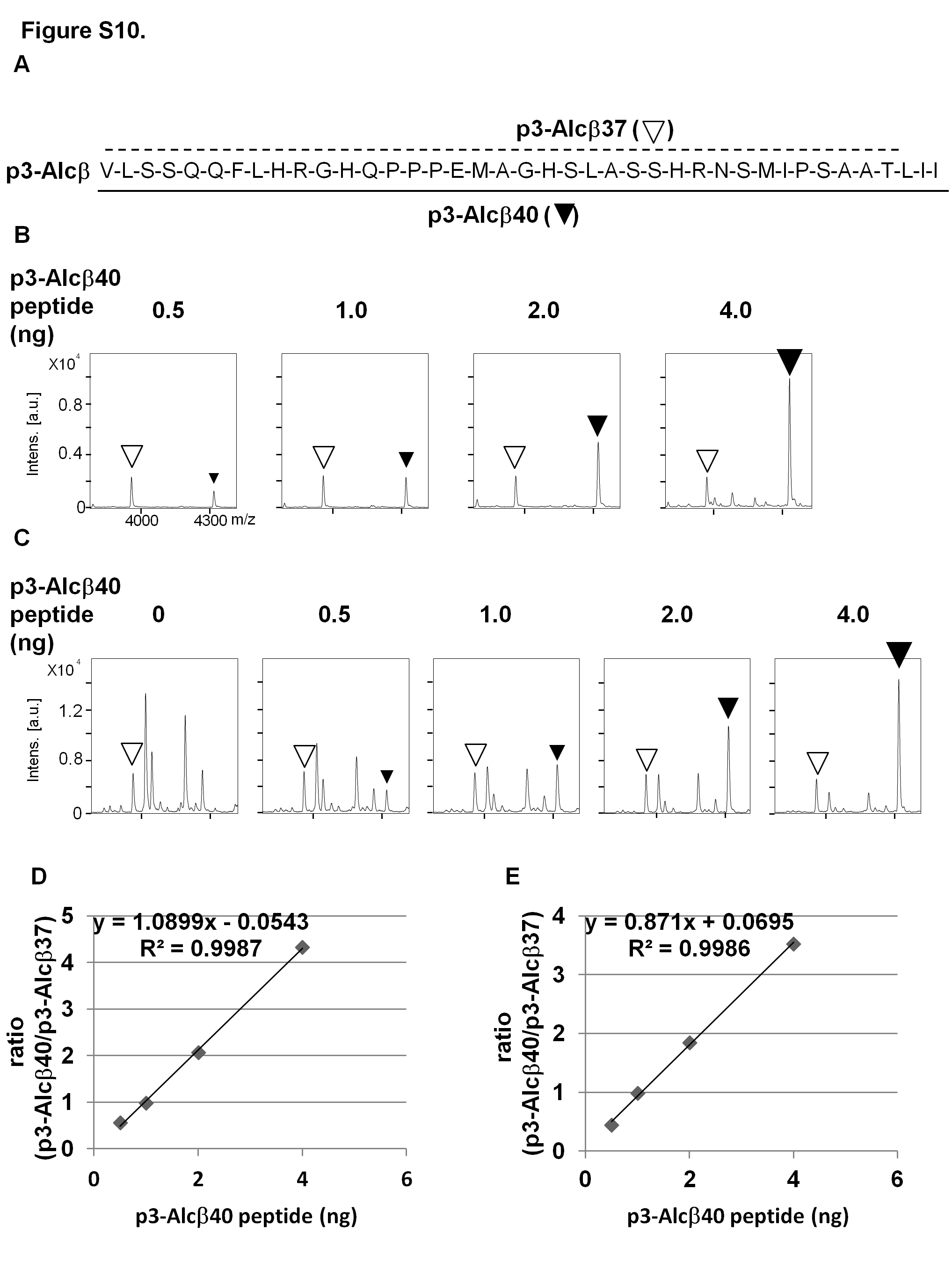

Supplement: Figure S10 — Quantitative accuracy of immunoprecipitation-mass spectrometric analysis in the presence of another peptide (II). Synthetic p3-Alcβ37 and p3-Alcβ40 were subjected to immunoprecipitation with UT143 and analyzed with MALDI-TOF/MS. A. Amino acid sequence of p3-Alcβ37 and p3-Alcβ40. B–C. Representative immunoprecipitation-mass spectra of synthetic p3-Alcβ peptides in PBS (2 mL) containing 0.1% (W/V) bovine serum albumin (B) or cultured medium (2 mL) of mock HEK293 cells (C). To the fixed amount (1 ng) of synthetic p3-Alcβ37 peptide (open arrowhead) indicated amount (0, 0.5, 1.0, 2.0 and 4.0 ng) of synthetic p3-Alcβ40 peptide (closed arrowhead) was added, and subjected to immunoprecipitation. The cells don't secrete p3-Alcβ species but show non-specific products as signals, which are detectable in C, but not in B, along with synthetic p3-Alcβ37 (open arrowhead) and p3-Alcβ40 (closed arrowhead). D–E. Quantitative accuracy of the ratio of p3-Alcβ40/p3-Alcβ37. The relationship of area ratios of p3-Alcβ40/p3-Alcβ37 with various amounts of synthetic p3-Alcβ40 peptide were analyzed (panel D indicates the result of B, and panel E indicates the result of C). The synthetic p3-Alcβ37 levels are not affected in the presence of increased amount of synthetic p3-Alcβ40 peptide (B) and unknown immunoprecipitates (C), and the p3-Alcβ40/p3-Alcβ37 ratio increased proportionally with the increased amount of synthetic p3-Alcβ40 peptide (R2 = 0.99866 in D and R2 = 0.99861 in E), indicating the quantification of a specific peptide is not affected in the presence of increased amounts of another peptide in this immunoprecipitation-mass spectrometric analysis. (TIF) [file pone.0062431.s010.tif]

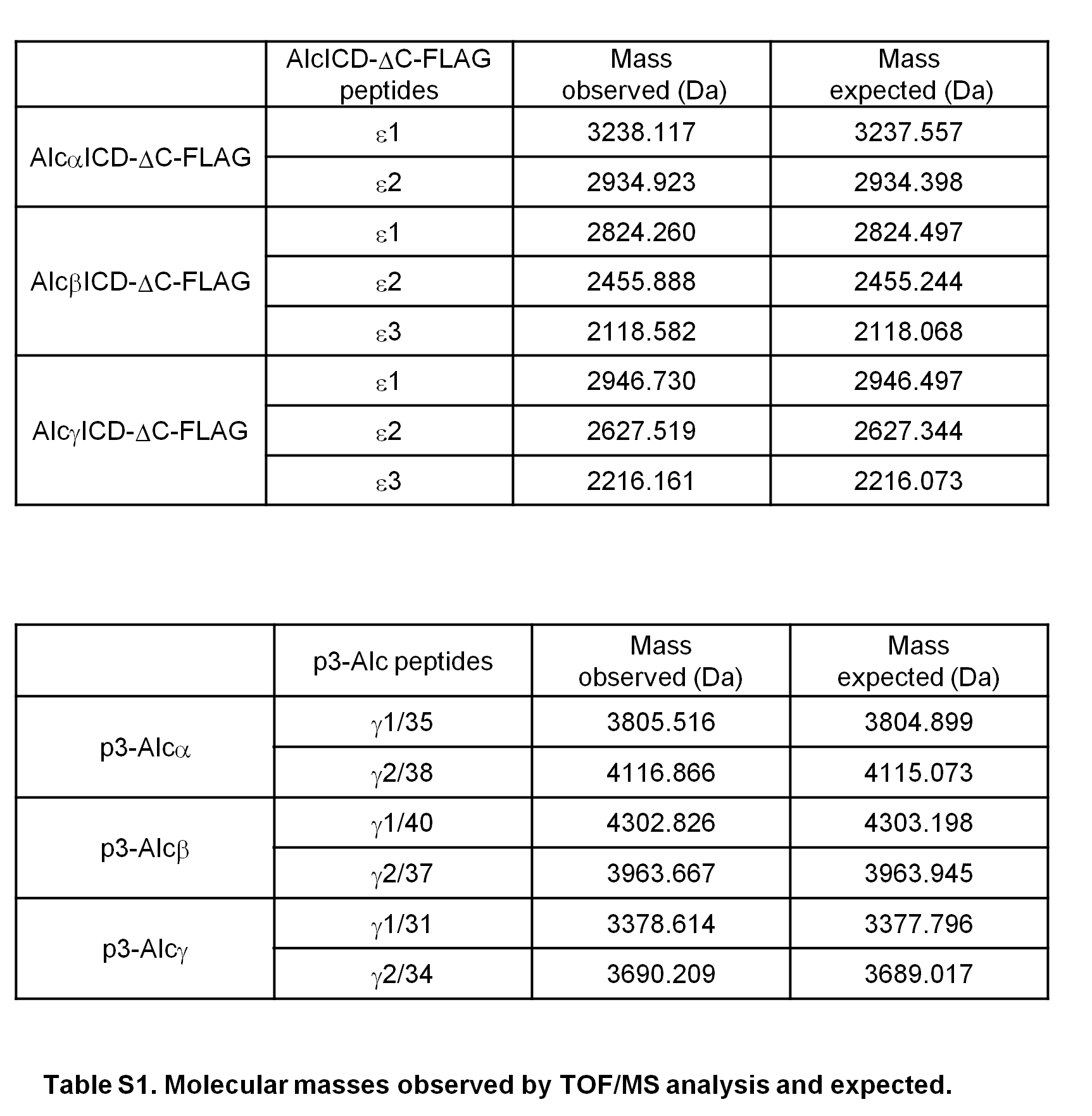

Supplement: Table S1 — Molecular masses observed by TOF/MS analysis and expected. Molecular masses (Da) of Alc ICD-ΔC-FLAG peptides (upper) and p3-Alcs (lower) generated by in vitro γ-secretase assay with cell membranes. The p3-Alcα peptide products γ1/35 and γ2/38 indicate p3-Alcα35 and p3-Alcα38, respectively, but not p3-Alcα2N+35 and p3-Alcα2N+38, which are secreted by cultured cells [17], because the in vitro γ-secretase assay with cultured cell membranes generates dominantly p3-Alcα species but not p3-Alcα2N+ species (see Fig. S4A). (TIF) [file pone.0062431.s011.tif]

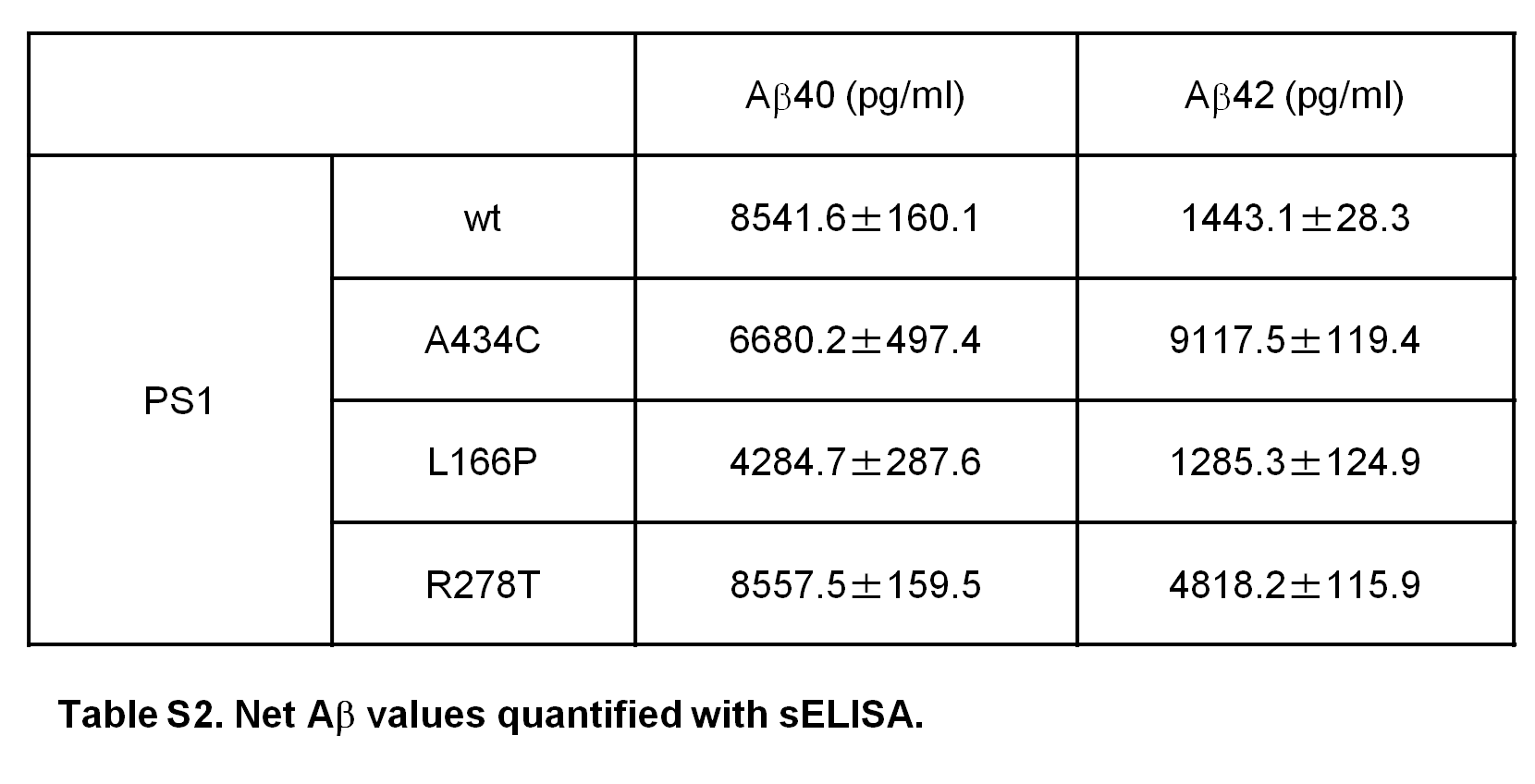

Supplement: Table S2 — Net Aβ values quantified with sELISA. Medium Aβ40 and Aβ42 values of the studies indicated in Fig. 2D were quantified with sELISA [27], and the average values are summarized with standard deviation (n = 4). (TIF) [file pone.0062431.s012.tif]
